# The Biological and Biomechanical Role of Transglutaminase-2 in the Tumour Microenvironment

**DOI:** 10.3390/cancers13112788

**Published:** 2021-06-03

**Authors:** Robert Tempest, Sonia Guarnerio, Rawan Maani, Jamie Cooper, Nicholas Peake

**Affiliations:** Biomolecular Sciences Research Centre, Sheffield Hallam University, Sheffield S1 1WB, UK; robert.tempest@shu.ac.uk (R.T.); s.guarnerio@shu.ac.uk (S.G.); rawan.a.maani@student.shu.ac.uk (R.M.); 19125250@brookes.ac.uk (J.C.)

**Keywords:** transglutaminase, biomechanics, extracellular matrix, tumour microenvironment

## Abstract

**Simple Summary:**

Transglutaminase-2 (TG2) is an enzyme primarily involved in protein cross-linking, which has been shown to play a role in the development and progression of numerous cancers. Increasing evidence indicates that TG2 is capable of modulating the tumour microenvironment (TME), resulting in changes which influence tumour initiation, growth, and metastasis. This review aims to highlight TG2’s role in the biological and biomechanical changes observed in the TME and the potential for therapeutic targeting of these changes in order to improve patient outcomes.

**Abstract:**

Transglutaminase-2 (TG2) is the most highly and ubiquitously expressed member of the transglutaminase enzyme family and is primarily involved in protein cross-linking. TG2 has been implicated in the development and progression of numerous cancers, with a direct role in multiple cellular processes and pathways linked to apoptosis, chemoresistance, epithelial-mesenchymal transition, and stem cell phenotype. The tumour microenvironment (TME) is critical in the formation, progression, and eventual metastasis of cancer, and increasing evidence points to a role for TG2 in matrix remodelling, modulation of biomechanical properties, cell adhesion, motility, and invasion. There is growing interest in targeting the TME therapeutically in response to advances in the understanding of its critical role in disease progression, and a number of approaches targeting biophysical properties and biomechanical signalling are beginning to show clinical promise. In this review we aim to highlight the wide array of processes in which TG2 influences the TME, focussing on its potential role in the dynamic tissue remodelling and biomechanical events increasingly linked to invasive and aggressive behaviour. Drug development efforts have yielded a range of TG2 inhibitors, and ongoing clinical trials may inform strategies for targeting the biomolecular and biomechanical function of TG2 in the TME.

## 1. Introduction

The enzyme Transglutaminase-2 (TG2), also known as tissue transglutaminase, is found in many different tissues, cell types, and subcellular compartments and has been shown to be associated with both normal cellular processes and various disease states. It is the most highly expressed member of the transglutaminase enzyme family, whose main catalytic activity is the Ca^2+^ dependent creation of lysine-glutamine isopeptide bonds, leading to protein cross-linking (transamidation). However, other enzymatic activities have also been linked to TG2, including deamidation and GTPase signalling, and each of these enzymatic functions has been studied in the context of cancer biology. TG2 structurally consists of four distinct globular domains: an N-terminal β-sandwich containing a fibronectin and integrin binding site; a domain containing a catalytic triad (Cys277, His335, and Asp358), primarily for acyl-transfer, as well as a conserved tryptophan; and two β-barrel domains, one containing a phospholipase C binding sequence and one containing the C-terminus [1,2,3,4]. The various activities and wide array of targets attributed to TG2 have led to the elucidation of a role in numerous cancers, linking to pathways involved in tumour initiation, progression, and eventual metastasis. In addition to mediating cancer cell behaviour and intracellular signalling, recent evidence also suggests TG2 is involved in alterations to the biomechanical environment and signalling in the tumour microenvironment (TME), and this review aims to highlight and discuss the impacts of these processes on tumour progression.

## 2. TG2 and the Hallmarks of Cancer

The wide range of subtypes, mutational backgrounds, and organs of origin emphasise that cancer is highly heterogenous at a genetic and molecular level. However, despite this diversity, the general principle of a progressive evolution of normal cells to a neoplastic state has been neatly conceptualised as a multistep acquisition of six key hallmarks of cancer [5,6]. These original hallmarks include sustaining proliferative signalling, evading growth suppressors, resisting cell death, enabling replicative immortality, inducing angiogenesis, and activating invasion. TG2 has a diverse range of substrates and is implicated in a number of processes linked to these hallmarks, including epithelial mesenchymal transition (EMT), cancer stem cell survival, drug resistance, inflammatory and proliferative signalling, and invasive and metastatic behaviour [7] (Table 1). Prominent TG2 expression has been identified in a diverse range of cancers including leukaemia, prostate cancer, breast cancer, renal cancer, lung cancer, ovarian cancer, glioblastoma, cervical cancer, colorectal cancer, squamous cell cancers, mesothelioma, and pancreatic cancer [8,9,10,11,12,13,14,15,16,17,18]. Whilst there is considerable evidence indicating a role for TG2 in the progression of these cancers, some studies also show involvement in tumour suppressive pathways [19]. This ability to exert contrasting effects may be dependent on structural conformation, with variation between transamidation and GTP-binding forms exhibiting different effects within the same cellular context [20].

### 2.1. Cellular Proliferative Signalling

The ability to sustain limitless proliferation is considered one of the key traits of cancer cells [48], and the presence of mitogenic growth signalling transitions cells from a quiescent state into active proliferation. Mitogenic signalling is modulated by a variety of molecules, including growth factors, components of the extracellular matrix (ECM), and inter-cell adhesive/interaction molecules [23]. TG2 is able to promote cellular proliferation and cell survival through its functional relationship with transforming growth factor-beta (TGF-β), a multifunctional cytokine involved in numerous processes including proliferation, differentiation, and immune function. TG2 expression is regulated by TGF-β via SMADs and TGF-β-activated kinase 1, leading to activation of the transcription factor nuclear factor-κB (NF-κB) and enhanced cellular proliferation, resulting in formation of spheroids and metastasis [21]. However, much like TG2, the role of TGF-β in cancer proliferation appears to be context-dependent, with studies showing both inhibition and promotion of cancer proliferation [49].

TG2 is also linked to cancer proliferation through other signalling routes. For example, Fu et al. [22] showed that TG2 knockdown impaired the proliferation of glioma stem cells, via reduced DNA binding 1 (ID1) protein expression. Proliferation was restored by overexpressing ID1, highlighting ID1 as a downstream mediator of TG2 via activation of the PI3K/AKT pathway. Moreover, TG2 has been associated with the accumulation of β-catenin, normally stimulated by activation of the Wnt pathway, which translocates to the nucleus and stimulates expression of CyclinD-1 and c-Myc, maintaining proliferation of ovarian cancer cells [23]. TG2 has also been observed to promote proliferation in gastric cancer via the extracellular signal-regulated protein kinases 1 and 2 (ERK1/2) pathway [24]. TG2 knockdown supressed cellular proliferation, and with the introduction of a specific ERK1/2 inhibitor, proliferation was partially reversed, suggesting an involvement of the ERK1/2 pathway in mediating TG2-driven proliferation.

### 2.2. Evading Growth Suppressors

The acquisition of sustained proliferation is complementary to the ability of cancer cells to evade growth suppression [50]. Cancer cells acquire the capability to circumvent the regulatory processes that negatively regulate cellular proliferation, which are mainly dependent on the action of tumour suppressors [51]. Two well-characterised tumour suppressor pathways involve retinoblastoma protein (RB) and tumour protein p53 (p53) [52]. These tumour suppressor genes function to either repress the cell cycle or promote apoptosis [53]. RB protein has been shown to play a pivotal role in the negative control of the cell cycle, whereas p53 activates expression of numerous genes regulating cell death, cell cycle arrest, senescence, and DNA repair [54,55].

TG2 activity has been recognised to modulate the activity of these tumour suppressors. In a hypophosphorylated state, RB inhibits cellular proliferation by modifying the functionality of transcription factors involved in regulating the expression of genes for transitioning from G1 to S phase in the cell cycle [56]. Several studies have shown that RB is a substrate for TG2 kinase activity [25], which mediates an anti-apoptotic effect by phosphorylation of RB. Earlier studies of TG2 also showed that RB was a substrate in lymphoma cells undergoing apoptosis and reported that TG2 protected RB from caspase-induced degradation in a transamidation-dependent manner [26].

TG2 expression also has an impact on the tumour suppressor p53. The Mouse double minute 2 protein (MDM2) binds and ubiquitinates p53 for degradation, and the ability of p53 to induce transcription of MDM2 generates a negative feedback system [57]. However, this negative feedback loop can be interfered with by the kinase activity of TG2, preventing the subsequent degradation and leading to the accumulation of p53, thereby facilitating potential further apoptosis [27].

### 2.3. Resisting Cell Death

Apoptosis is a form of programmed cell death which results in the orderly removal of damaged cells through a caspase-dependent mechanism, and avoiding this fate, despite extensive mutational damage, is a key characteristic in cancer development [58]. This also provides a challenge to treatment, as many chemotherapy approaches rely on triggering intrinsic or extrinsic apoptotic cell death [59]. Intriguingly, several studies have highlighted the role of TG2 and its involvement in the apoptotic process. Early observations that increased TG2 expression accompanied the apoptotic programme [60] were followed by studies observing proapoptotic or antiapoptotic effects that are fundamentally dependent on its cellular context and structural conformation [61]. For example, induction of TG2 activity using the calcium ionophore A23187 results in high levels of apoptosis in cancer cells [62,63], and there is evidence that TG2 crosslinking of the transcription factor Sp1 can induce apoptosis [64]. By contrast, multiple studies have demonstrated an inhibition of apoptosis by TG2, through mechanisms involving modification of caspase-3 and Bax activities [28,29,30,31], underlining the importance of cellular context for a complex system of TG2-mediated influence. Indeed, intracellular localisation and TG2 isoform both appear to be critical factors in determining how TG2 mediates cell fate [65,66], and TG2 itself is a target of caspase-3 [67]. In fact, multiple variants of TG2 have been identified, and the effects and impacts of this complex regulation remain to be clarified [68]. The involvement of TG2 in the efferocytosis of apoptotic cells [69,70] and in cross-linking during necrosis [71] suggests an important role in containing tissue damage and restricting potentially damaging inflammation as a result of cell death.

The activation of tumour necrosis factor-related apoptosis-inducing factor (TRAIL) eradicates cancer cells via the activation of the extrinsic apoptosis pathway and through ligation to receptors including death receptor 4 (DR4)/TRAIL-R1 and DR5/TRAIL-R2 [72]. By establishing an acquired TRAIL resistance in lung cancer cells, Li et al. [32] identified TG2 as one of the most highly upregulated genes via gene expression screening, and inhibition led to sensitization and apoptosis [33]. The introduction of Epidermal Growth Factor Receptor (EGFR)-mediated activation of extracellular-signal-regulated kinase (ERK) and c-Jun N-terminal kinase (JNK), which increased TG2 expression, contributed to the acquired resistance of TRAIL and a reduction of MMP-9, a matrix metalloproteinase involved in invasion and migration. EGFR therefore appears to be a fundamental upstream signalling pathway of TG2 in cells with TRAIL resistance [32].

### 2.4. Chemoresistance

The development of drug resistance in cancer cells presents a major clinical challenge to successful cancer treatment. Understanding the fundamental mechanisms of drug resistance is therefore essential for the application of anti-cancer therapeutics. Interestingly, the selective expression of TG2 in cancer cells has been demonstrated to promote chemoresistance through a number of mechanisms. This phenomenon was first observed by Mehta [73], who demonstrated that a doxorubicin-resistant subclone of breast cancer cells expressed higher levels of TG2 than doxorubicin-sensitive cells. The ability of TG2 to modulate chemoresistance in a range of cancer types has since been reported in several different studies, and chronic expression of TG2 triggers a range of signalling pathways that contribute to the development of drug resistance [10]. Along with extrinsic TRAIL resistance, doxorubicin and cisplatin resistance has been reported in a range of cancer cell types, through interaction with the pathways that lead to intrinsic resistance to apoptosis. More recent studies also point to the TG2 mediated resistance to a newer generation of treatment approaches, such as those aimed at mechanistic target of rapamycin complex 1 (mTORC1) [74,75] and histone deacetylase inhibitors [76], showing that TG2 is involved in a wide spectrum of chemoresistance mechanisms.

The self-degradative process of autophagy is a fundamental cellular homeostasis program, essential for balancing sources of energy at critical times of development and nutrient stress [77]. Cellular conditions that induce an autophagic response include oxidative stress, hypoxia, nutrient deprivation, and exposure to chemotherapeutics [78]. As the activities of TG2 and autophagy can both be induced under cellular stress, including chemotoxic stress, and as both are linked to chemoresistance, a potential role for TG2 in mediating autophagy has been explored. For instance, overexpression of TG2 in mantle cell lymphoma activates NF-κB signalling to increase STAT3 and IL-6 signalling, which leads to an enhanced autophagy-dependent cell survival response [79]. Furthermore, the autophagic response has a positive feedback impact on IL-6 and TG2 signalling, further stimulating this survival mechanism [80]. Work in the context of cystic fibrosis has demonstrated that in response to endoplasmic reticulum (ER) stress TG2 can cause cross-linking and aggregation of beclin-1, a protein which plays a key role in autophagy [81] through the regulation of autophagosome formation, with impacts linked to the inflammatory profile of this disease. Given the relationship between ER stress, reactive oxygen species (ROS), and cancer [82,83], and the inflammatory signalling pathways mediated by NF-kB and IL-6, further work to explore the context-dependant contribution of TG2 in cancer progression would be informative.

### 2.5. Enabling Unlimited Replicative Immortality

An additional hallmark of cancer is the capability for unlimited replicative potential, in contrast to the behaviour of non-cancer cells, which are restricted by the Hayflick limit [84]. This trait is closely connected to the hallmarks already described: insensitivity to antigrowth and apoptotic signals and growth signal autonomy, leading to the dysregulation of mitogenic signalling [5]. The majority of cancers are adenocarcinomas, arising within epithelial layers. Epithelial layers are defined by their polarity and adhesion to neighbouring cells and the ECM, and loss of these attachments triggers anoikis, leading to apoptosis [85]. The EMT is a phenotypic switch allowing cells to avoid this fate through the downregulation of adhesion molecules such as e-cadherin, and reprogramming from an epithelial to a mesenchymal gene expression profile and upregulation of survival transcription factors, including NF-kB and regulators of cell adhesion, such as Zeb1, Zeb2, Slug, TWIST, and SNAIL. Accordingly, cancer cells frequently adopt EMT to confer cell survival characteristics and avoid anoikis during invasive and metastatic progression [86]. TG2 has been shown to confer survival from anoikis in a manner dependent on fibronectin binding [87], and a number of studies have identified a role for TG2 in EMT. TG2-induced NF-kB activation promotes the EMT–CSC phenotype in tumour cells, and TGF-β exhibited a complete dependency on TG2 for its capability for inducing EMT in breast cancer cells [35]. Moreover, the introduction of TG2-siRNA prior to TGF-β treatment failed to induce EMT, further highlighting TG2 as a downstream mediator of TGF-β-induced EMT in mammalian epithelial cells [36]. TG2-induced EMT has also been associated with further phenotypic stem cell properties, such as self-renewal capability and cell plasticity [37]. This association highlights the ability of TG2, not only to induce EMT within the initial tumour, but perhaps to also contribute to stemness-associated properties within secondary tumours, and thereby support metastasis.

There is growing evidence that the indefinite growth potential of many tumours is sustained by this population of cancer cells showing characteristics of a de-differentiated stem cell phenotype, and that these cancer stem cells (CSCs) are linked to TG2 activity [34]. CD44 is a transmembrane glycoprotein commonly used as a marker for CSCs of many solid malignancies and plays an essential role in tumour progression by supporting proliferation and enabling replicative immortality, metastasis, and chemoresistance through the activity of several signalling pathways [88,89]. Interestingly, TG2 has been shown to increase CD44 activity in breast cancer cells, regulating the promotion of stem cell phenotype and metastatic potential [90], and inhibition of TG2 lead to depleted CSC surface antigens such as CD44 in renal cell carcinoma [45]. Recently, it was also demonstrated by Condello et al. [91] that functional inhibition of TG2 fibronectin-binding supressed complex formation, CSCs survival, and stemness-associated with Wnt/β-catenin signalling. This suggests an additional function of TG2 in CSC phenotype, spheroid proliferation, and tumour-initiating capacity modulated through the direct interaction with Wnt receptor Frizzled 7 (Fzd7).

## 3. TG2 as a Key Functional Player in the TME

### 3.1. The Tumour Microenvironment

**Figure 1:**
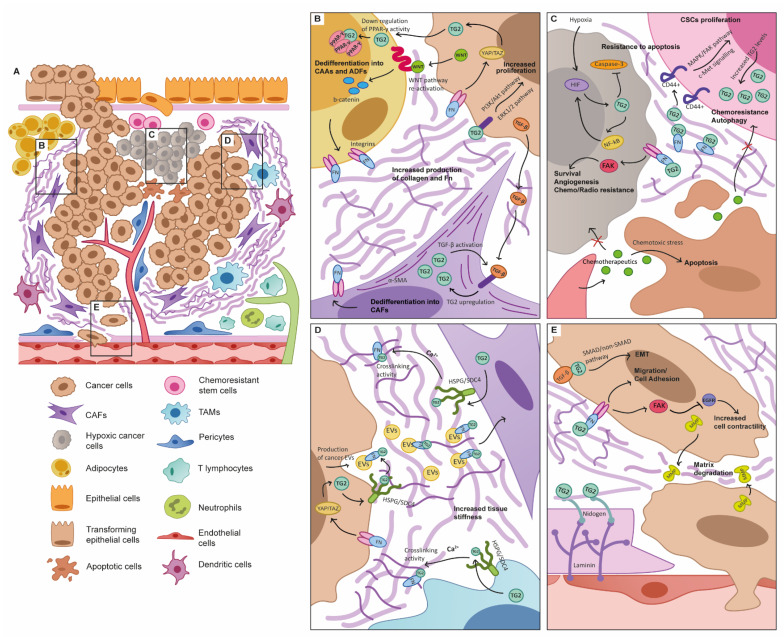
TG2 roles in the tumour microenvironment (TME). (**A**) Schematic representation of the tumour microenvironment (TME) showing the presence of resident and infiltrated cellular constituents and extracellular matrix (ECM) components. Cancer, stroma, and immune system constantly interact, and within this complex system TG2 plays a role in multiple pathways and responses. Subfigures B-E are magnifications of the indicated areas within the TME. (**B**) TG2 is linked to adipocyte and fibroblast phenotype. Upregulation of cancer-derived TG2 may inhibit adipogenesis by cross-linking of proliferative-activated receptor y (PPARγ) along with other events, such as the re-activation of WNT canonical pathway. The TGF-β/TG2 link is associated with the activation of fibroblasts to a de-differentiated, contractile cancer-associated fibroblast (CAF) phenotype. Increased stromal stiffening enhances integrin activation and signalling through pathways such as YAP/TAZ which can further regulate TG2 expression. (**C**) TG2 in chemoresistance, apoptosis, and replicative immortality. Chemotherapeutics induce apoptosis in sensitive cancer cells, but in chemoresistant cells several mechanisms are activated to contrast death. A hypoxic-induced loop between HIF/TG2/NF-κB is responsible for apoptotic resistance in cancer cells. Increased expression of TG2 promotes chemoresistance and survival in cancer cells through several pathways but also through the binding with integrins and the activation of FAK signalling. TG2 increases CD44+ activation, leading to the high proliferative and chemo-resistant cancer stem cell (CSC) phenotype. (**D**) TG2 cross-linking activity contributes to TME stiffening of the primary and metastatic sites. TG2 linked to fibronectin (FN) upon HSPG/SDC4 release from fibroblasts and macrophages in the extracellular space activates its cross-linking activity, remodelling the ECM and contributing to cancer cells migration and fibrosis. Cancer-derived extracellular vesicles (EVs) express TG2/FN on their membranes and alter the metastatic niche through paracrine signalling. (**E**) TG2 is linked to cancer invasive behaviour. The reciprocal relationship between TGF-β and TG2 mediates epithelial-to-mesenchymal transition (EMT) in cancer cells, leading to enhanced migration, which is also increased by the binding TG2/Fn/integrins and the following adhesion to the ECM. Increased cell contractility is promoted by downstream signalling of TG2/Fn/integrins binding, such as the inhibition of EGFR activity through FAK signalling. The balance between TG2 enzymatic activity on laminin/nidogen complexes and upregulation of matrix metalloproteinases (MMPs) by cancer cells contributes to basement membrane (BM) integrity, and thus invasive potential.

The original definition of the “hallmarks” of cancer acknowledged that in addition to rapidly proliferating cancer cells that resist death, cancer progression is fundamentally dependant on the interaction of tumours with their environment, defining processes such as angiogenesis and invasion which lead to, and are dependant on, cellular and tissue remodelling of their surroundings. This was refined in the updated hallmarks [6], to reflect the observations that during all the stages of carcinogenesis, malignant cells interact with the environment around them, creating an effective organ-like system that is defined as the tumour microenvironment (TME). In this complex system, cellular and non-cellular components cross-talk with the tumour and in some cases become functionally altered by the malignant cells and the unique metabolic and molecular conditions, with an increase in chromosomal instability being shown in TME stromal cells [92,93,94]. This can lead to host tissue cellular components becoming dysfunctional in support of cancer progression [95]. A number of studies have identified a strong expression and activity of TG2 in the stromal tissue surrounding tumours [47,96,97], and proposed TG2 as a biomarker with stromal expression showing distinct clinical profiles and prognosis when compared to epithelial expression [98,99]. Since differential expression of the multiple variants of TG2 is dependent on cell type and disease state [68], this points towards a critical and complex role within this multi-cellular TME (Figure 1).

### 3.2. TG2 and the Cancer-Associated Fibroblast

Fibroblasts are a major contributor to the tumour supportive conditions found in the TME. Under normal physiological conditions, fibroblast function in the stroma is the synthesis and deposition of ECM components. In this normal fibroblast functionality, TG2 contributes to cell-matrix interactions by targeting key ECM components, including collagen and fibronectin, and plays a role in the regulation of cell spreading, migration, and reorganisation of the ECM [100]. Fibroblasts play a major role in wound healing and fibrosis, becoming activated and displaying a highly contractile, myoblastic phenotype. This activation state appears to be mirrored in the TME, where highly abundant fibroblasts promote tumour growth and invasive potential. These aberrant cells are known as cancer-associated fibroblasts (CAFs) and often characterised by smooth muscle actin (SMA) and vimentin expression, and induce significant desmoplasia and remodelling of the ECM. TG2 is closely involved with the wound healing response [101,102], appears functionally involved in the development of the activated myofibroblast phenotype [103] linked to development of fibrotic disease, and has recently been proposed as an additional marker of CAFs, shown to be upregulated in comparison to normal fibroblasts both in patient samples and in fibroblasts activated by TGF-β [104].

These CAFs display a diverse range of phenotypes, potentially due to their different lineages and mechanisms of activation. The population appears to include activated resident stromal fibroblasts, as well as dedifferentiated pericytes, adipocytes, endothelial cells, mesenchymal stem cells, and epithelial cells. This leads to the heterogeneity observed in various studies, which showed diverse fibroblast subpopulations such as inflammatory, quiescent, antigen-presenting, and myofibroblasts, as well as their precursor forms [105,106,107]. Senescent fibroblasts have also been identified in the TME [108], a phenotype linked to increased accumulation of transglutaminase-crosslinked products [109]. Their mechanisms of activation are also diverse, with roles in fibroblast growth factor (FGF), platelet-derived growth factor (PDGF), ROS, TGF-β, and tumour necrosis factor (TNF) [110].

Of these factors, TGF-β appears to be particularly important. Cancer-secreted TGF-β is capable of inducing CAF phenotype [111,112,113], and TGF-β signalling in CAFs is closely linked to poor clinical outcome [94,114], with inhibition leading to remodelling of CAF dynamics, better immune response, and disease regression in in vitro models. The well-established link between TG2 and TGF-β is therefore critical, with TGF-β upregulating TG2, while TG2 is also known to be capable of transforming inactive TGF-β to its active form [115,116,117].

CAFs can also promote EMT in resident cancer cells. This transformation is caused by paracrine signalling from the TME, and it has been demonstrated that CAF-induced EMT was in fact dependent on TG2 expression mediated by IL-6 in hepatocellular carcinoma [118], positioning TG2 as critical for the cancer/CAF cross-talk within the TME.

### 3.3. TG2 and Adipocyte Function

Obesity is recognised as a major risk factor for many cancers and is characterised by an increase in adipocyte cell size and number, with subsequent expansion of adipose tissue. Obesity contributes directly to activation of myofibroblasts and increased desmoplasia, and obesity and metabolic syndromes are linked to expression of TG2 [119,120]. Cancer associated adipocytes (CAAs) are adipocytes found in the TME and have been shown to be present in a number of cancers including breast [121], pancreatic [122], and colorectal [123]. CAAs exhibit a dedifferentiated phenotype, and the crosstalk between CAAs and tumour cells can eventually give rise to the formation of adipocyte-derived fibroblasts (ADFs) following activation of the Wnt signalling pathway by tumour cells. This results in upregulation of type I collagen and fibronectin, increased invasiveness and migratory potential, and increased expression of CAF fibroblast specific markers [124]. TG2 has been shown to be an inhibitor of adipogenesis [125], providing a potential link to the transformation of normal adipocytes to a dedifferentiated CAA phenotype. In fact, TG2 deficient mouse embryonic fibroblasts have been shown to exhibit increased lipid accumulation, increased expression of adipogenic transcription factors PPARγ and C/EBPa, and absence of Pref-1/Dlk1 and defective canonical Wnt signalling, all involved in the inhibition of adipogenesis and maintenance of the preadipocyte phenotype [125]. Exogenous TG2 was shown to reverse these effects, suggesting that cancer-cell derived TG2 may be capable of having the same effect. Proliferator-activated receptor-γ (PPARγ) is a known target of TG2, and cross-linking causes aggregation and downregulation of PPARγ activity [81,126]. While this has been shown in the context of inflammation in cystic fibrosis and celiac disease, there may be a role for this mechanism in the TME, since PPARγ is critical for adipocyte maturation and CAA/ADF balance.

### 3.4. TG2 and the Immune System in Cancer

The interactions between inflammation, immune response, and cancer are closely connected to progression and outcome [6]. Despite the presence of TG2 in inflammatory signalling observed in conditions as diverse as cystic fibrosis [126], celiac disease [127], fibrosis [128], and sepsis [129], a possible triangular relationship between immune system–tumour–TG2 remains relatively unexplored. The positive feedback loop originating from the TG2–TGF-β association is a strong pro-inflammatory signal, and a similar loop characterises the relationship between TG2 and NF-kB, the central transducer of inflammatory signalling. TG2 mediates signalling via NF-kB, by either polymerization of the inhibitory I-kBa subunit or inducing phosphorylation of RelA/p65 [130,131], and is also reciprocally regulated by an NF-kB binding motif in its promoter region [132].

Production of the pro-inflammatory cytokine Intereukin-6 (IL-6) in the TME has been established to be dependent on TG2 expression [133,134]. IL-6 production has been associated with the acquisition of a stem cell phenotype, as well as involvement in the EMT [135]. This IL-6 expression was dependant on TG2 upregulated through NF-kB, PI3K-, and JNK-dependent signalling cascades in promoting a cancer stem cell phenotype and inducing the EMT and metastasis when amplified by Interleukin 1 beta (IL1B) production [90].

Within the TME, many cancers have significant immune infiltrate, and yet malignant cells are able to avoid and exhaust this immune response, even in the presence of extensive inflammatory signalling [136]. It is clear that macrophages have a critical role to play in the tumour microenvironment, and tumour-associated macrophages (TAMs) are the prominent immune cell observed in the TME and clearly linked to disease progression [137]. There is evidence from studies of CNS inflammation that TG2 can mediate macrophage recruitment [138], and this appears to be an early event in many cancers. Macrophages can differentiate into M1 and M2 lineages, with the former associated with pro-inflammatory functions and the latter linked to immune resolution [139]. TAMs are frequently imbalanced towards the M2 phenotype, and it is known that TG2 is a marker of this differentiated lineage [140]. In multiple sclerosis, it has been shown that TG2 is a key mediator in macrophage differentiation and myelin phagocytosis, with a reduction in TG2 expression pushing macrophages towards an M1, pro-inflammatory state [141]. The relation between syndecan-4 and TG2 on the surface of macrophages is significant, as it seems to support the recruitment and migration against apoptotic or unfunctional cells, ultimately protecting from a chronic inflammatory state [142]; and extracellular release performed through syndecan-4 promotes TG2 to contribute to cross-linking of the ECM, which is a contributor to fibrosis [143]. The ECM of the TME bears a lot of the hallmarks of a fibrotic response, with dense collagen deposition and cross-linking [144,145]. The link between TAMs, ECM remodelling in cancer [146], and TG2 is currently under-explored, and given the growing evidence that TAM/CAF-mediated remodelling appears critical to the exclusion of anti-cancer immune responses [147], further work in this field would be informative. TG2 is also associated with dendritic cell function [148], notably in the interaction between dendritic cells and T-cells [149,150], which could have significant implications within the TME, where T-cell exhaustion is frequently linked to poor outcome, and where driving anti-cancer T-cell responses is a major focus of immunotherapy [151,152]. Notably, TG2 expression has recently been linked to immunosuppression in pancreatic cancer, correlating with upregulation of immunomodulatory cells and exclusion of effector cells, in mechanisms involving NF-kB and the regulation of programmed death ligand-1 (PD-L1) [153], and TG2 has been identified in an immune signature associated with poor prognosis in lung squamous cell carcinoma [99].

### 3.5. The Hypoxic TME

Like all cells, cancer cells require the exchange of oxygen and nutrients, as well as the ability to evacuate metabolic waste via the production of tumour-associated neovasculature via angiogenesis [154]. Rapid proliferation of cancer cells is linked to aberrant metabolic activity and a dependence on aerobic glycolysis, which leads to a characteristic production of lactate and acidic conditions in the TME [155,156]. TG2 appears to be capable of influencing mitochondrial activity and metabolic profile [157,158], and intriguingly providing a link between metabolism and ECM remodelling [159]. An interesting feature of glutaminases is the release of ammonia as a reaction biproduct, and there is evidence that this may be a mechanism by which cancer cells tolerate acidic conditions [160], though this may not actually result in a net change in pH, due to an amine being required as a substrate during transamidation. The intratumoral vessels of the TME are often incompletely formed with irregular architecture characterised with increased fenestration and “leakiness” [161]. Restricted blood supply to a rapidly expanding tumour leads to hypoxic areas, and Hypoxia-inducible factor-1 (HIF-1) expression has been correlated with poorer clinical outcomes, as it confers resistance to apoptosis of tumour cells [30]. Under normal conditions HIF-1α protein is expressed but unstable and oxygen is required for it to be hydroxylated. Under hypoxic conditions HIF-1a accumulates and translocates to the nucleus, binding to HIF-1β to express HIF-1 [162], and the anti-apoptotic role of TG2 is further mediated by HIF-1, which inhibits the main executioner of apoptosis in both intrinsic and extrinsic death signalling caspase 3 [30,163]. Studies on mesothelioma models also indicate that hypoxia induces TG2 via HIF2 [16], and through inhibitor experiments it is clear that TG2 activity plays an important role in the survival of hypoxic environments in response to the HIF family of transcription factors.

### 3.6. Induction of Angiogenesis

The process of tumour-associated angiogenesis occurs through the continual activation of the angiogenic switch, subsequently triggering normal quiescent vasculature to alternate to sustained angiogenesis, which supports the requirements of a developing tumour [164]. Hypoxia leads to the release of vascular growth factors, such as vascular endothelial growth factor (VEGF), which are produced to stimulate angiogenesis, and resident vascular cells such as pericytes and endothelial cells are also fundamental for cancer progression [165]. The presence of pericytes has been shown to protect against the evolution of disease; endothelial cells are essential for generating new vessels due to the hypoxic-induced growth factors produced by the cancer cells [165,166].

TG2 is abundantly distributed in endothelial cells and has been shown to exert effects on tubule formation, resulting in inhibition of angiogenesis and cancer progression [167]. However, Wang et al. [40] showed that the site-directed irreversible inhibition of TG2 transamination activity resulted in inhibition of angiogenesis by regulation of VEGF release into the ECM, ultimately facilitating activation of signalling via VEGF receptor 2. More recently Lei et al. [42] demonstrated that inhibiting TG2 GTP-binding activity led to suppression of the downstream NF-κB/HIF1α pathways, ultimately leading to inhibition of angiogenesis. In renal cell carcinoma, TG2 has also been shown to promote angiogenesis through degradation of p53, which leads to HIF1α activation and increased production of VEGF [41]. The fundamental ability of TG2 to support or supress angiogenesis is most likely governed by its cellular context and structural conformation.

Mechanical alterations across the vascular wall have an impact on angiogenesis. Compression of the blood vessels in the TME reduces blood flow leading to hypoxia, and thus contributing to cancer progression [168]. TG2 has been shown to alter vascular stiffness through regulation of smooth muscle cell contractility and proliferation [169], as well as through matrix remodelling; altering the mechanical properties of collagen fibres by cross-linking in the vascular wall [43]. Changes in mechanical properties have been shown to influence drug delivery and immunotherapy interventions, with angiotensin receptor blockers such as losartan showing promise through the targeting of tension in the TME and promotion of vascular function [170,171]. Moreover, the depletion of CAFs with an inhibitor of the sonic hedgehog pathway alleviated solid stress, decompressed blood and lymphatic vessels, and increased perfusion, leading to more effective therapies [168].

## 4. TG2 Is Key to the Biomechanical Progression of Cancer

### 4.1. The Mechanics of Cancer Progression

As is evident from the impact of mechanical stress on vascular function and cancer treatments, it has become increasingly apparent that cancer progression is not only dependant on the cellular physiology, but also on the biophysical and biomechanical properties of the TME [172], driven by the unique cellular composition and a dense, remodelled ECM, which plays an integral role in the mechanical progression of the tumour [173]. Dysregulation of fibrous proteins (such as collagen), adhesive glycoproteins (such as laminins), and proteoglycans (such as heparan sulphate proteoglycans) [174] are all linked to the hallmarks of cancer, and there is growing evidence of their contribution to abnormal physical attributes, including increased solid stresses, elevated interstitial fluid pressure, altered matrix architecture, and increased tissue stiffness of the TME [175].

Solid stresses are mechanical forces (tensile, compressive, and shear) that are generated due to the uncontrolled growth of the tumour cells, remodelling of the ECM, and the mechanical confinement by the surrounding ECM [176]. These forces have a direct impact on the proliferation and migration of tumour cells [177] and activate CAFs, which in turn further promote the migration of tumour cells [178]. CAFs generate contractile forces on the ECM, which is known to result in the release and activation of TGF-β1 [179], and activation of TGF-β signalling in fibroblasts, which in turn upregulates various cross-linking enzymes in the ECM, including TG2 that has a further impact on the biomechanical environment by increasing tissue stiffness [96]. Mechanical stresses can also directly induce and stabilize an open catalytically active conformation of TG2; hence, active TG2 results in a cross-linked ECM that can act as a storage depot for TGF-β [180].

Increased tissue stiffness is the most well-observed mechanical abnormality in tumours, it contributes to several biological tumour processes, including proliferation [172,181], angiogenesis [182], invasion [183], and metastasis [184]. Tumour tissues tend to become stiffer as the tumour progresses, due to several factors, such as increased matrix deposition, cross-linking by lysyl oxidase (LOX) and TG2 enzymes [185,186], matrix remodelling, and the accumulation of both solid and interstitial pressures [187]. Various studies have correlated the activity of CAFs to matrix stiffness, and it has been shown that CAFs induce collagen cross-linking, leading to stiffer ECM; the increased ECM stiffness and TGF-β signalling in turn activates fibroblasts, generating a positive feedback loop that further promotes the ECM stiffening [188]. The relationship between CAFs, TG2, and TGF-β indicates that TG2 is likely to be a vital contributor to this matrix-stiffness positive feedback loop.

### 4.2. Mechanical Forces and Invasion Initiation

Mechanical forces are vital for the initiation of the invasion-metastatic cascade [177]. Throughout all steps of the cascade, mechanical interactions between the invasive tumour cells and the surrounding TME seem to be involved, as invading cells respond to the mechanical modulations in the TME and alter the mechanical properties of their microenvironment to promote their progression and invasion [189]. For example, in epithelial cancers, where cells migrate collectively [190], the mechanical compressive stress triggers the initiation of the invasion, and cells can undergo phenotypic transformation when compressed and become leader cells at the leading edge, which can coordinate collective cell migration as they extend protrusions towards the direction of migration and guide other migratory “follower” cells [191]. Accordingly, TG2 has been found to facilitate invasion at the leading edge, and it has been shown that EGF stimulates the expression of TG2 at the leading edge through Ras and c-Jun N-terminal kinase pathways, resulting in the enhanced motility and invasiveness of tumour cells [44]. Mechanical signalling mechanisms also contribute to the induction of EMT, supporting cancer cells to adopt motile phenotypes and detaching from the primary site [192]. Indeed, physio-mechanical mechanisms are not only involved in the initiation of invasion but are also needed to facilitate the migration of invasive cells through different microenvironments (stroma, blood vessel endothelium, vascular system, and secondary tissue site). For instance, the adhesion strength of tumour cells to their surrounding stroma determines whether they can detach and migrate through the surrounding tissues and barriers [193], while it is evident that TG2 is involved in the adhesion and attachment of tumour cells, due to its association with the integrins linking cells to the ECM [194]. In addition, elastic deformations of tumour cells are needed during the intravasation and extravasation processes to facilitate the penetration of cells through the endothelial cell–cell junctions, and TG2 likely plays a role in this, since it is linked to the intracellular tension of cancer cells through the loss of EGFR-mediated inhibition of cell contractility [195]. Lastly, the interplay between cell velocity and adhesion through the vascular system determines the location of the malignant tumour’s secondary site [189].

### 4.3. Interaction and Invasion through the Basement Membrane

Following the mechanical induction of invasion, invading tumour cells must migrate across the basement membrane (BM); the ECM barrier separating the epithelium and the stroma in nearly all tissues. They are mainly composed of collagen IV [196], laminin, and glycoproteins (nidogen, heparan sulphate proteoglycans) [197]. The BM’s biomechanical properties provide structural and adhesion support to cells and harbour various growth factors that contribute to cell growth, survival, and migration, such as TGF-β, heparin epidermal growth factor (HB-EGF), FGF, and VEGF [198]. The density of the covalently cross-linked network of the BM protects the cells from the surrounding mechanical stress and acts defensively to prevent invading cells from reaching the stroma [199].

However, mechanical compromise and the proteolytic remodelling mechanisms of the BM facilitate the breaching of the BM and the crossing of tumour cells into the stroma [200,201]. Matrix metalloproteinases (MMPs), a subgroup of the metalloproteinase gene family, play a key role in the BM’s proteolytic remodelling and ECM degradation [202], and are notably upregulated in invasive carcinomas [203]. MMPs act prominently to degrade ECM proteins in opposition to the crosslinking and mechanical strengthening provided by TG2 to resist proteolytic attack [204]. For instance, TG2 mediates the binding between laminin and nidogen. Laminin is the most abundant non-collagenous protein of the BM that is highly susceptible to proteolytic degradation, and its binding to nidogen protects it from degradation. Hence, TG2 is crucial for the stability of the BM [205,206].

It therefore appears that TG2 is expressed as a host response to invasion [207]. TG2 treatment restricts the invasive behaviour of tumour cells [208], and knockout of TG2 leads to enhanced tumour metastasis [209]. Indeed, in vivo injection of cDNA TG2 plasmids resulted in a reduction in the number of metastatic foci, and thus a better prognosis [210]. An increase in collagen density coupled with TG2 activity in 3D tumour/stroma co-culture models appeared to limit the growth of cancer spheroids [96], which agrees with other studies showing that a stiffer ECM restricts the rate of growth of cancer cells in vitro. However, this may be counter-productive in the long-term, as cancer cells respond to stiffer microenvironments through enhanced integrin activation and more invasive behaviour [211]. Whilst initially restricting growth, stiffer matrices promote higher expression of Mena, an invadopodium protein, and Fibronectin (FN), which are then associated with cell migration [183]. The consequences of cross-linking and matrix stiffening supported by TG2 may therefore be context- and stage-dependant.

Whilst the activity of TG2 in restricting invasion through cross-linking and proteolytic resistance appears to be in contrast to MMPs in the integrity of the BM, studies revealed a complex relationship between both proteins. It was observed that TG2 activates the expression of MMP-2 [212], with a coordinated interplay with MT1-MMP, which hydrolyses TG2 at the tumour/stroma boundary. However, the cross-linking activity of TG2 at the early stages of the tumour, together with deposition of collagen and an increased stiffness around the perimeter of the tumour, may act as a barrier protecting against invasive behaviour [47,96,213]. It was shown that TG2-cross-linked collagen is more resistant to MMP’s proteolytic degradation [214]. Hence, the loss of the TG2 at the tumour–stromal interface alters matrix modulation and decreases the cell–matrix interaction, which in turn reduces adhesion and promotes cell motility and invasion [215,216], and it is intriguing that a number of studies identify down-regulation of TG2 in association with invasion and metastasis [47,217,218].

### 4.4. Role of TG2 in Interstitial Extracellular Matrix Remodelling

Pathological remodelling of the ECM, which is altered by tumour-induced interactions, is a hallmark of cancer and fibrotic diseases [219]. Tumours, in association with other cells in the TME, can shape their microenvironment to promote their progression and migration; they deposit, biochemically and biophysically modify, and degrade the tumour-associated ECM. ECM proteins, which are often known to provide structural support, play a key role in the cell signalling pathways providing biochemical signals that are interpreted by cell surface receptors [220], such as integrins [221]; thus, initiating the cell signalling cascades that are involved in vital cellular processes, such as proliferation [220].

The interstitial matrix forms a porous network surrounding the cells that connects stromal cells. In contrast to the BM, the matrix is remodelled in response to mechanical forces. In tumours, the matrix remodelling alters various biophysical and biochemical mechanisms that have an impact on ECM stiffness, cell signalling, tumour progression, and migration [172,222,223]. The remodelling process is highly regulated however, and cells dysregulate this process in pathological conditions such as tissue fibrosis and cancer [224]. In particular, the increased ECM matrix stiffness due to cellular deposition and cross-linking mechanisms seems to develop a tumorigenic ECM that facilitates tumour progression.

The vital role of TG2 in matrix remodelling is not only achieved through its cross-linking function, but also through its role in the mechano-activation of CAFs and tumour-associated macrophages, which play a vital role in the production and remodelling of collagen and other ECM proteins; with a central role for TG2, as discussed earlier [186,225]. While, activated fibroblasts are the major producer of the interstitial matrix, secreting ECM proteins such as fibronectin (FN) and collagen [226] and exerting contractile forces supporting tumour growth and progression [227].

TG2 contributes to the matrix stiffness and is involved in the maintenance of the mechanical homeostasis of the ECM. The degradation of ECM by the MMPs promotes ECM synthesis and deposition by fibroblasts, and the matrix stiffness is then adjusted by fibroblasts through the secretion of TG2 and LOXs [228]. These homeostatic feedback mechanisms are altered in the tumour due to the altered expression levels of ECM proteins (such as collagens) and ECM-modifying enzymes (such as LOXs and TG2) leading to stiffer ECM [47,211,229]. In contrast to the restriction of invasion medited by TG2 [47,96,208,209,210,215,216], overexpression of TG2 in breast cancer is associated with increased cell migration, metastasis, recurrence, and poor overall survival which may be due to the cross-linked stroma [97,211].

### 4.5. Role of TG2 in Biomechanical Signalling of TME

The interplay between the mechanics and biomechanics of TME mediates tumour progression. For instance, mechanical forces induced by tumour growth trigger stromal cells to release various growth factors that assist with tumour progression. Notably, TG2-induced matrix modulation is correlated to the induction of various biomechanical signalling pathways. It has been shown that TG2-induced matrix stiffening drives integrin clustering to enforce the focal adhesions and intracellular growth signalling pathways, such as the PI3K [214,230,231] and Hippo pathways [186]. The Hippo pathway, Yes-associated protein (YAP) and transcriptional coactivator with PDZ-binding motif (TAZ), is one of the oncogenic signalling pathways that is activated due to TG2-induced matrix stiffening and enriched stroma with cross-linked collagen and active fibroblasts, promoting cell proliferation and tumour progression [187]. In fact, the cross-talk between TG2 activity and the YAP/TAZ pathway appears bi-directional, with growing evidence that TG2 is a target gene for YAP/TAZ as well as a driver of EMT, migration and invasion through the YAP1/TEAD transcription complex [38,39,232]. Moreover, the TG2-induced modulation of focal adhesions and the subsequent activation of FAK were linked to the increased contractility of tumour cells [195], and to chemo-resistance phenotype due to the activation of several downstream signalling pathways, including the apoptosis-resistance mechanism [233].

TG2 has many vital roles beyond protein cross-linking, e.g., it mediates the non-enzymatic protein–protein interactions that are involved in the cell-matrix crosstalk. In contrast to the observations linking the cross-linking activity of TG2 to restricted tumour invasion [47,96,208,209,210,215,216], TG2-induced biomechanical alterations can also promote tumour invasion. Surface TG2 has been shown to act as a bridge between the α1 and α3 integrins families and fibronectin. This activity, which is enabled by the strong affinity of TG2 for the 42-kDa fragment of fibronectin, has a role in cell adhesion and migration [234,235]. A higher level of TG2 in tumours results in an accumulation of FN [236] and increased association of integrins (integrin- β1, β4, and β5), with FN leading to enhanced cell adhesion. Whereas, the loss of TG2 suppressed integrins interaction with FN, cell attachment, migration, and invasion since it compromised the integrin-FN association [45,194,237,238]. Therefore, inhibition of TG2 can be considered as a potential therapeutic strategy.

In addition to the integrin-FN association, TG2 has been shown to promote invasion through the activation of RAC (GTPase) signalling protein that mediates cell movement through its involvement in the structural changes of the actin cytoskeleton, thus controlling cell migration [239]. TG2 contributes to actin fibre assembly through the induction of RAC [12]. Indeed, the loss of TG2 has shown an impact on the assembly of actin stress fibre [45].

## 5. TG2—A Stage-Specific Cancer Target

### 5.1. TG2 and Tumour Metastasis

Most patients who die from cancer do so because it has metastasised from the primary site [240]. Given the complex multicellular events that mediate tumour growth within specific microenvironmental niches, the processes leading to the formation of the metastatic niche are only beginning to be understood, and there is evidence of a key role for TG2 in these processes; with studies suggesting a pro- and anti-metastatic functions through differential expression in metastatic cancers, cell lines, and consequences of inhibition in animal models across a range of cancer types [17,47,134,209,210,241,242,243,244].

As Paget’s classic “seed and soil” hypothesis implies, the formation of a metastatic niche is widely thought to occur prior to the arrival of an invading tumour, and the growing interest in the field of extracellular vesicles (EVs) has drawn attention to their role in this process. EVs are small, membrane-enclosed packages of bioactive molecules which are released by cells and can travel through biofluids to be taken up by recipient cells, both locally and distally. TG2 has been linked to their biogenesis and has been shown to be a key cargo, which is transported in its active form [245,246]. Release of TG2 as a cargo is stimulated by TGF-β and dependent upon the interaction with syndecan-4 [200]. EVs are also heavily enriched in regulatory RNA molecules such as miRNA, a number of which have been shown to regulate TG2, both theoretically and experimentally [247].

As EVs can be released from cancer cells, enter the circulation, and reach all parts of the body, they are heavily implicated in the creation of the pre-metastatic niche [248] and appear to be involved in organotropism; determining the eventual site of metastasis in an integrin-dependent manner [249]. The interaction between TG2 and fibronectin appears to be important, and in this context, TG2 binding fibronectin has been found on the surface of MVs secreted by cancer cells that reach the secondary organ, raising interest in their possible influence on modification of cell adhesion in the stroma of the pre-metastatic niche [245,250]. Although their presence has been demonstrated and linked to poor prognosis, the specific mechanism of action is still not fully understood. However, the direct secretion of microvesicles derived from cancer cells and the uptake and functional activity of TG2 in recipient fibroblast cells has been demonstrated, resulting in the activation of mitogenic signalling and subsequent transformation [245]. This was dependent on the simultaneous transfer of fibronectin, which is cross-linked within the microvesicle and is required for fibroblast activation [245,250]. The enrichment of TG2 in MVs following h-RAS-induced EMT provides a potential link between EVs and the ability of TG2 to mediate EMT and subsequent invasive behaviour [251].

Since TG2 plays an important role in mediating cell tension and contractility, with implications for cancer cell motility [100], and as cell stiffness has also been linked to cell uptake capability [252], there could be great interest in fully characterising TG2, EVs, and cancer progression and whether these mechanisms could directly feed into the influence of EVs on cancer progression and the cell substrates and enzymatic functions involved. Actin and tubulin have both been implicated in TG2-mediated processes [253,254,255], potentially providing a link between matrix and cytoskeletal mechanics.

Indeed, cellular mechanics are likely to be relevant throughout the TME, and, interestingly, the uptake of EVs and the subsequent activation of CAFs is linked to matrix stiffness, providing a potential link between TG2, matrix mechanics, EVs, and cellular behaviours driving cancer [256]. However, EVs are not the only method proposed for TG2 externalisation, with export via the P2X7 purinergic receptor also identified [257]; a system also involved in cancer progression and metastasis [258].

### 5.2. Targeting Mechanobiology of TME

**Figure 2:**
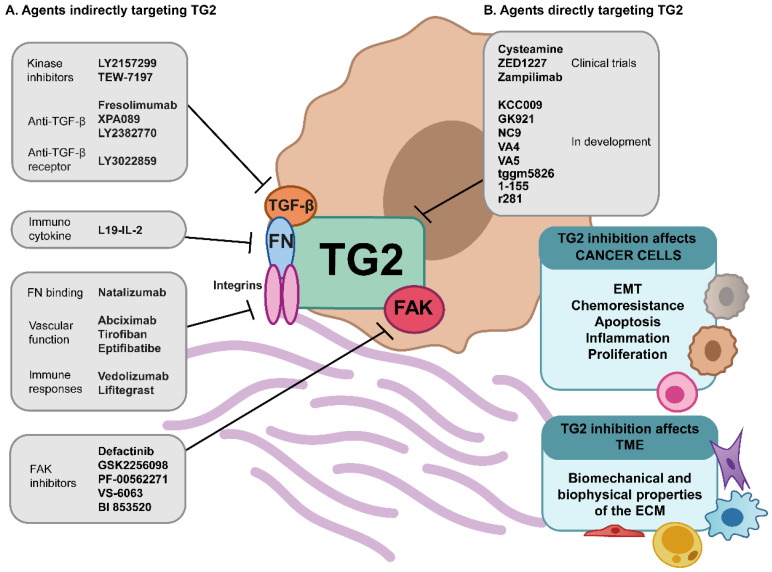
The potential impacts of direct and indirect targeting of TG2-linked biological and biomechanical processes in the TME. (**A**) Recent drug development efforts in the field of biomechanotherapeutics have established several clinical leads targeting molecular events driving the altered biomechanical properties of the TME. Many of these promising candidates target mediators that interact with TG2, pointing to indirect effects on TG2 function. These include agents targeting TGF-β, including kinase inhibitors such as LY2157299 (Galunisertib) and TEW-7197, anti-TGF-β antibodies (Fresolimumab, XPA089, LY2382770), and anti TGF-β-receptor antibodies (LY3022859). Agents targeting fibronectin species within the TME are developed to improve immune responses (L19-IL-2). Integrins are another key target, with 6 agents progressing through various clinical pipelines, including natalizumab (targeting fibronectin binding of α4β1 and α4β7), agents targeting vascular function (abciximab, tirofiban, Eptifibatibe), and agents targeted to immune responses (vedolizumab targeting α4β7 and Lifitegrast targeted at αLβ2), with many other integrin-targeting candidates in pre-clinical stages. Focal adhesion kinase (FAK) is also a candidate target, with Defactinib, GSK2256098, PF-00562271, VS-6063, and BI 853,520 showing promise in fibrotic and cancer indications. (**B**) Direct targeting of TG2 is a viable approach to targeting the TME; cysteamine, ZED1227, and Zampilimab are TG2-targeted agents all either validated or in clinical trials for various indications, with a number of promising candidates showing pre-clinical efficacy in cancer models, such as KCC009, GK921, NC9, VA4, VA5, TGGM5826, 1-155 and R281. The impact of direct or indirect effects on TG2 activity includes altering chemosensitivity, apoptosis, inflammatory and proliferative signalling, EMT, and stem cell characteristics across a wide range of cancer cell types, and also wider effects in the TME, particularly the biomechanical and biophysical properties of the cross-linked, remodelled ECM and subsequent biomechanical signalling, with impacts on the cellular milieu associated with cancer development and progression.

The growing evidence pointing towards the importance of biophysical and biomechanical factors in driving the hallmarks of cancer has resulted in a number of therapeutic strategies being developed. The critical role and therapeutic potential of TG2 in the pathological remodelling of ECM and altered mechanical properties of tumour and stroma are demonstrated by its interaction with a number of strategies currently in development as cancer treatments (Figure 2).

Stiffness-activated FAK has been linked to the activation and survival of myofibroblasts and cancer cells and is considered a potent target in fibrosis. A FAK inhibitor, Defactinib, has shown a potent anti-fibrotic effect in fibrosis and desmoplastic tumours in preclinical models and is now being investigated in humans [259]. Trials of the agents GSK2256098, PF-00562271, VS-6063, and BI 853,520 are also underway and so far show good safety profiles, although with limited efficacy; but with potential for combination therapy [260]. TGF-β1, which has a clear reciprocal relationship with TG2, is another potent mechanical target. A range of approaches have been developed to block the TGF-β signalling cascade, with key clinical leads in cancer treatment including anti-TGF-β antibodies, anti-TGF-β receptor antibodies, and small molecular inhibitors of TGFR-linked kinases [261]. Integrins are also highly desirable targets, due to their key role in mediating biomechanical signalling in cancer. Recent reviews of this field identified some 430 clinical leads, but only seven that had reached the clinical market (abciximab; tirofiban; eptifibatide; natalizumab; vedolizumab; and lifitegrast), indicating potential limitations in isolating anti-cancer effects of integrin signalling [262]. Strategies to target the relationship between integrins and other mediators such as TGF-β or fibronectin are also under evaluation [263], and TG2 remains an under-explored addition to this route.

Compelling evidence now shows that ECM matrix stiffness is correlated to tumour progression and metastasis, reducing tumour vascularization, and drug delivery to tumours. Therefore, targeting matrix crosslinking and stiffness is a promising treatment approach [264]. Accordingly, TG2 and LOXs have been identified as mechano-therapeutic targets to reduce matrix stiffness and prevent the mechano-activation that promotes tumour progression [265]. In fibrosis, a non-specific LOX inhibitor β-aminopropionitrile (BAPN) has been shown to reduce tissue stiffness and alleviate fibrosis [266]. While in cancer, KCC009 selective inhibitor of TG2 blocked the remodelling of fibronectin in ECM, and sensitized tumours to chemotherapy due to the remodelling of fibronectin in ECM [267].

### 5.3. Current State of TG2 Inhibitors

The concept of targeting TG2 to treat cancer has a long history, resulting in significant drug development efforts. TG2 conformation is key in determining the effects it confers and this also has ramifications in therapeutic targeting. The GTP signalling, or “closed”, form is the most prevalent in the intracellular environment, where high levels of GTP are seen, and the transamidase active, or “open”, form is more prevalent in the extracellular space, though it also can be preferred in the presence of high calcium levels. It has been suggested that GTP signalling/closed conformation contributes to pro-survival signalling pathways and cancer cell survival, and that transamidase/open conformation serves to cross-link and sequester tumour suppressors, as well as modify the ECM to benefit the tumour [7].

One way in which treatment may be targeted is by modulating the conformation of TG2, and treatment with TG2 inhibitor NC9 in immortalised normal mouse cells resulted in a shift to the open configuration [268]. This affect has also been shown with TG2 inhibitors VA4, an VA5 in epidermal squamous cell carcinoma cells [269], and importantly was shown to prevent both GTP signalling and transamidase activity by also blocking the transamidase site, as well as forcing a confirmation that restricts the GTP binding site. Due to the calcium binding nature of the transamidase/open form, treatments which increase intracellular calcium levels are also capable of forcing increased activation of transamidase activity, leading to cancer cell death [46,270].

Giving the increased evidence of the impact of TG2 on various signalling proteins, blocking the TG2 binding to signalling protein is another promising approach. For example, the TG2 inhibitor, GK921, has been shown to be effective in a xenograft model in renal cell cancer (RCC), the GK921-binding site overlaps with that of p53 and thus prevents it from binding to TG2, and therefore maintains the stability of the tumour suppressor activity of p53, resulting in a significant anticancer effect. In addition, GK921 inactivates the enzyme by binding to its N-terminus, resulting in a conformational change that increases the non-covalent self-polymerisation of the protein, resulting in the inactivation of the enzyme [271].

Some drugs targeting TG2 are already being developed for the treatment of other diseases. Cysteamine is an established, commercially available, safely tolerated inhibitor of TG2, initially developed for the treatment of cystinosis but which has since shown promise in the treatment of other diseases such as Huntington’s chorea, Leigh disease, mitochondrial disease, Parkinson’s disease, and Rett syndrome, and it is now being considered for re-evaluation in new disease contexts including cancer, given the growing knowledge of the role of TG2 in the TME [272]. ZED1227 developed by Zedira is a TG2 inhibitor developed for the treatment of celiac disease and is already in advanced clinical trials [273]. Zedira are currently also moving towards TG2 inhibitors for fibrosis, which may open up the future possibility of re-purposing towards TME applications. UCB Celltech has also developed the inhibitory antibody Zampilimab, which is in phase I/II trails for adult kidney transplant recipients with chronic allograft injury. These pharmacological agents demonstrate contrasting approaches to TG2 targeting. Small, cell-permeable inhibitors are likely to have significantly different impacts on the TME context compared to antibody-based agents targeted at matrix cross-linking in the extracellular environment. Targeting of ECM cross-linking has been trialled before in the treatment of fibrosis. Simtuzumab is a humanised monoclonal antibody that targets LOXL2, a catalyst in the cross-linking of collagen. Unfortunately, clinical trials were halted at phase 2 due to a lack of efficacy [274]. It is promising however that TG2 shows potential in this context as an ECM targeting, anti-fibrotic agent [128], and development of small molecule inhibitors as anti-fibrotic agents continues [103].

### 5.4. Future Directions

There already exists a huge weight of data demonstrating the presence and activity of TG2 in the TME, in addition to the growing mechanistic understanding of its role in mediating cancer progression at the cellular and extracellular levels. Functioning in both driving cell behaviour within the complex TME milieu, as well as exerting ECM cross-linking and mediating biophysical and biomechanical forces, TG2 remains a viable target as a cancer treatment. Drug development efforts have yielded a range of inhibitory approaches, some of which are clinically validated, and which could be re-purposed as either standalone treatments or adjuvants to existing cancer treatments. Lessons could be learned from trials on MMP inhibitors, where disappointing results were partly linked to drug trials designed to target stages of disease that were unresponsive to inhibition of proteolytic activity [275]. The growing understanding of the dynamic role of TG2 as cancer evolves and progresses, coupled to the enormous efforts characterising the mechanical events driving cancer cell survival and invasion, suggest that pre-clinical development focussed on an appropriate window of opportunity has the potential to position TG2 as a valid, useful, biological, and biomechanical target to expand the options available for the treatment of cancers, particularly advanced-stage tumours that show extensive ECM deposition and remodelling, and that currently have poor prognosis.

## Figures and Tables

**Table 1:** TG2 and the hallmarks of cancer. The various mechanisms and signalling pathways shown to be linked to TG2 activity in the hallmarks of cancer.

Hallmark	Key Mechanisms/Pathways	References
Sustaining proliferative signals	TGF-β, PI3K/AKT, Β-catenin/Wnt, ERK1/2	[21,22,23,24]
Evading growth suppressors	Regulation of RB/p53 pathways	[25,26,27]
Resisting cell death	Caspase-3/Bax, TRAIL	[28,29,30,31,32,33]
Enabling replicative immortality	CSCs (CD44), EMT, YAP/TAZ	[34,35,36,37,38,39]
Inducing angiogenesis	VEGF, NF-κB/HIF1α, ECM remodelling	[40,41,42,43]
Activating invasion	EGF, EMT/TGF-β, type I collagen/β-1 integrins, Rac, ECM alterations	[12,35,36,37,44,45,46,47]

## References

[1] Murthy S.N.P., Iismaa S., Begg G., Freymann D.M., Graham R.M., Lorand L. (2002). Conserved Tryptophan in the Core Domain of Transglutaminase is Essential for Catalytic Activity. Proc. Natl. Acad. Sci. USA.

[2] Király R., Demény M., Fésüs L. (2011). Protein Transamidation by Transglutaminase 2 in Cells: A Disputed Ca^2+^-Dependent Action of a Multifunctional Protein. FEBS J..

[3] Hwang K.C., Gray C.D., Sivasubramanian N., Im M.J. (1995). Interaction Site of GTP Binding Gh(Transglutaminase II) with Phospholipase C. J. Biol. Chem..

[4] Cerione R.A., Liu S., Clardy J. (2004). Structural Basis for the Guanine Nucleotide-Binding Activity of Tissue Transglutaminase and its Regulation of Transamidation Activity. Proc. Natl. Acad. Sci. USA.

[5] Hanahan D., Weinberg R.A. (2000). The Hallmarks of Cancer. Cell.

[6] Hanahan D., Weinberg R. (2011). Hallmarks of Cancer: The Next Generation. Cell.

[7] Eckert R.L. (2019). Transglutaminase 2 Takes Center Stage as a Cancer Cell Survival Factor and Therapy Target. Mol. Carcinog..

[8] Pierce A., Whetton A.D., Meyer S., Ravandi-Kashani F., Borthakur G., Coombes K.R., Zhang N., Kornblau S. (2013). Transglutaminase 2 Expression in Acute Myeloid Leukemia: Association with Adhesion Molecule Expression and Leukemic Blast Motility. Proteomics.

[9] Han A.L., Kumar S., Fok J.Y., Tyagi A.K., Mehta K. (2014). Tissue Transglutaminase Expression Promotes Castration-Resistant Phenotype and Transcriptional Repression of Androgen Receptor. Eur. J. Cancer.

[10] Huang L., Xu A., Liu W. (2015). Transglutaminase 2 in Cancer. Am. J. Cancer Res..

[11] Park M.J., Baek H.W., Rhee Y., Lee C., Park J.W., Kim H.W., Moon K.C. (2015). Transglutaminase 2 Expression and its Prognostic Significance in Clear Cell Renal Cell Carcinoma. J. Pathol. Transl. Med..

[12] Lee H., Huang C., Chen W., Tsai C., Chao Y., Liu S., Chen J., Wu Y., Lee Y. (2018). Transglutaminase 2 Promotes Migration and Invasion of Lung Cancer Cells. Oncol. Res..

[13] Satpathy M., Cao L., Pincheira R., Emerson R., Bigsby R., Nakshatri H., Matei D. (2007). Enhanced Peritoneal Ovarian Tumor Dissemination by Tissue Transglutaminase. Cancer Res..

[14] Ayinde O., Wang Z., Pinton G., Moro L., Griffin M. (2019). Transglutaminase 2 Maintains a Colorectal Cancer Stem Phenotype by Regulating Epithelial-Mesenchymal Transition. Oncotarget.

[15] Fisher M.L., Adhikary G., Xu W., Kerr C., Keillor J.W., Eckert R.L. (2015). Type II Transglutaminase Stimulates Epidermal Cancer Stem Cell Epithelial-Mesenchymal Transition. Oncotarget.

[16] Zonca S., Pinton G., Wang Z., Soluri M.F., Tavian D., Griffin M., Sblattero D., Moro L. (2017). Tissue Transglutaminase (TG2) Enables Survival of Human Malignant Pleural Mesothelioma Cells in Hypoxia. Cell Death Dis..

[17] Verma A., Guha S., Diagaradjane P., Kunnumakkara A.B., Sanguino A.M., Lopez-Berestein G., Sood A.K., Aggarwal B.B., Krishnan S., Gelovani J.G. (2008). Therapeutic Significance of Elevated Tissue Transglutaminase Expression in Pancreatic Cancer. Clin. Cancer Res..

[18] Gundemir S., Monteagudo A., Akbar A., Keillor J.W., Johnson G.V.W. (2017). The Complex Role of Transglutaminase 2 in Glioblastoma Proliferation. Neuro Oncol..

[19] Tabolacci C., de Martino A., Mischiati C., Feriotto G., Beninati S. (2019). The Role of Tissue Transglutaminase in Cancer Cell Initiation, Survival and Progression. Med. Sci..

[20] Chhabra A., Verma A., Mehta K. (2009). Tissue Transglutaminase Promotes or Suppresses Tumors Depending on Cell Context. Anticancer Res..

[21] Cao L., Shao M., Schilder J., Guise T., Mohammad K.S., Matei D. (2011). Tissue Transglutaminase Links TGF-Β, Epithelial to Mesenchymal Transition and a Stem Cell Phenotype in Ovarian Cancer. Oncogene.

[22] Fu J., Yang Q., Sai K., Chen F., Pang J.C.S., Ng H., Kwan A., Chen Z. (2013). TGM2 Inhibition Attenuates ID1 Expression in CD44-High Glioma-Initiating Cells. Neuro Oncol..

[23] Condello S., Cao L., Matei D. (2013). Tissue Transglutaminase Regulates Β-catenin Signaling through a c-Src-dependent Mechanism. FASEB J..

[24] Wang X., Yu Z., Zhou Q., Wu X., Chen X., Li J., Zhu Z., Liu B., Su L. (2016). Tissue Transglutaminase-2 Promotes Gastric Cancer Progression Via the ERK1/2 Pathway. Oncotarget.

[25] Boehm J.E., Singh U., Combs C., Antonyak M.A., Cerione R.A. (2002). Tissue Transglutaminase Protects Against Apoptosis by Modifying the Tumor Suppressor Protein p110 Rb. J. Biol. Chem..

[26] Oliverio S., Amendola A., Di Sano F., Farrace M.G., Fesus L., Nemes Z., Piredda L., Spinedi A., Piacentini M. (1997). Tissue Transglutaminase-Dependent Posttranslational Modification of the Retinoblastoma Gene Product in Promonocytic Cells Undergoing Apoptosis. Mol. Cell. Biol..

[27] Mishra S., Murphy L.J. (2006). The p53 Oncoprotein is a Substrate for Tissue Transglutaminase Kinase Activity. Biochem. Biophys. Res. Commun..

[28] Yamaguchi H., Wang H.-G. (2005). Tissue Transglutaminase Serves as an Inhibitor of Apoptosis by Cross-Linking Caspase 3 in Thapsigargin-Treated Cells. Mol. Cell. Biol..

[29] Cho S., Lee J., Bae H., Jeong E.M., Jang G., Kim C., Shin D., Jeon J., Kim I. (2010). Transglutaminase 2 Inhibits Apoptosis Induced by Calcium-Overload through Down-Regulation of Bax. Exp. Mol. Med..

[30] Jang G., Jeon J., Cho S., Shin D., Kim C., Jeong E.M., Bae H.C., Kim T.W., Lee S., Choi Y. (2010). Transglutaminase 2 Suppresses Apoptosis by Modulating Caspase 3 and NF-kappaB Activity in Hypoxic Tumor Cells. Oncogene.

[31] Yoo J., Lim Y., Kim Y., Ha K. (2012). Transglutaminase 2 Promotes both Caspase-Dependent and Caspase-Independent Apoptotic Cell Death Via the Calpain/Bax Protein Signaling Pathway. J. Biol. Chem..

[32] Li Z., Xu X., Bai L., Chen W., Lin Y. (2011). Epidermal Growth Factor Receptor-Mediated Tissue Transglutaminase Overexpression Couples Acquired Tumor Necrosis Factor-Related Apoptosis-Inducing Ligand Resistance and Migration through C-FLIP and MMP-9 Proteins in Lung Cancer Cells. J. Biol. Chem..

[33] Frese-Schaper M., Schardt J.A., Sakai T., Carboni G.L., Schmid R.A., Frese S. (2010). Inhibition of Tissue Transglutaminase Sensitizes TRAIL-Resistant Lung Cancer Cells through Upregulation of Death Receptor 5. FEBS Lett..

[34] Eckert R.L., Fisher M.L., Grun D., Adhikary G., Xu W., Kerr C. (2015). Transglutaminase is a Tumor Cell and Cancer Stem Cell Survival Factor. Mol. Carcinog..

[35] Agnihotri N., Kumar S., Mehta K. (2013). Tissue Transglutaminase as a Central Mediator in Inflammation-Induced Progression of Breast Cancer. Breast Cancer Res..

[36] Kumar A., Xu J., Brady S., Gao H., Yu D., Reuben J., Mehta K. (2010). Tissue Transglutaminase Promotes Drug Resistance and Invasion by Inducing Mesenchymal Transition in Mammary Epithelial Cells. PLoS ONE.

[37] Kumar A., Gao H., Xu J., Reuben J., Yu D., Mehta K. (2011). Evidence that Aberrant Expression of Tissue Transglutaminase Promotes Stem Cell Characteristics in Mammary Epithelial Cells. PLoS ONE.

[38] Fisher M.L., Kerr C., Adhikary G., Grun D., Xu W., Keillor J.W., Eckert R.L. (2016). Transglutaminase Interaction with α6/β4-Integrin Stimulates YAP1-Dependent ΔNp63α Stabilization and Leads to Enhanced Cancer Stem Cell Survival and Tumor Formation. Cancer Res..

[39] Fisher M.L., Adhikary G., Kerr C., Grun D., Eckert R.L. (2017). Transglutaminase 2 Is a Direct Target Gene of YAP-TAZ-Response. Cancer Res..

[40] Wang Z., Perez M., Caja S., Melino G., Johnson T.S., Lindfors K., Griffin M. (2013). A Novel Extracellular Role for Tissue Transglutaminase in Matrix-Bound VEGF-Mediated Angiogenesis. Cell Death Dis..

[41] Lee S., Kang J.H., Ha J.S., Lee J., Oh S., Choi H., Song J., Kim S. (2020). Transglutaminase 2-Mediated p53 Depletion Promotes Angiogenesis by Increasing HIF-1α-p300 Binding in Renal Cell Carcinoma. Int. J. Mol. Sci..

[42] Lei Z., Chai N., Tian M., Zhang Y., Wang G., Liu J., Tian Z., Yi X., Chen D., Li X. (2018). Novel Peptide GX1 Inhibits Angiogenesis by Specifically Binding to Transglutaminase-2 in the Tumorous Endothelial Cells of Gastric Cancer. Cell Death Dis..

[43] Steppan J., Bergman Y., Viegas K., Armstrong D., Tan S., Wang H., Melucci S., Hori D., Park S.Y., Barreto S.F. (2017). Tissue Transglutaminase Modulates Vascular Stiffness and Function through Crosslinking-Dependent and Crosslinking-Independent Functions. J. Am. Heart Assoc..

[44] Antonyak M.A., Li B., Regan A.D., Feng Q., Dusaban S.S., Cerione R.A. (2009). Tissue Transglutaminase is an Essential Participant in the Epidermal Growth Factor-Stimulated Signaling Pathway Leading to Cancer Cell Migration and Invasion. J. Biol. Chem..

[45] Bagatur Y., Ilter Akulke A.Z., Bihorac A., Erdem M., Telci D. (2017). Tissue Transglutaminase Expression is Necessary for Adhesion, Metastatic Potential and Cancer Stemness of Renal Cell Carcinoma. Cell Adhes. Migr..

[46] Tabolacci C., Lentini A., Mattioli P., Provenzano B., Oliverio S., Carlomosti F., Beninati S. (2010). Antitumor Properties of Aloe-Emodin and Induction of Transglutaminase 2 Activity in B16–F10 Melanoma Cells. Life Sci..

[47] Cellura D., Pickard K., Quaratino S., Parker H., Strefford J.C., Thomas G.J., Mitter R., Mirnezami A.H., Peake N.J. (2015). miR-19-Mediated Inhibition of Transglutaminase-2 Leads to Enhanced Invasion and Metastasis in Colorectal Cancer. Mol. Cancer Res..

[48] Feitelson M.A., Arzumanyan A., Kulathinal R.J., Blain S.W., Holcombe R.F., Mahajna J., Marino M., Martinez-Chantar M.L., Nawroth R., Sanchez-Garcia I. (2015). Sustained Proliferation in Cancer: Mechanisms and Novel Therapeutic Targets. Semin. Cancer Biol..

[49] Ikushima H., Miyazono K. (2010). TGFβ Signalling: A Complex Web in Cancer Progression. Nat. Rev. Cancer.

[50] Gutschner T., Diederichs S. (2012). The Hallmarks of Cancer: A Long Non-Coding RNA Point of View. RNA Biol..

[51] Lowe S.W., Evan G., Cepero E. (2004). Intrinsic Tumour Suppression. Nature.

[52] Zhu L., Lu Z., Zhao H. (2014). Antitumor Mechanisms when pRb and p53 are Genetically Inactivated. Oncogene.

[53] Sherr C.J. (2004). Principles of Tumor Suppression. Cell.

[54] Giacinti C., Giordano A. (2006). RB and Cell Cycle Progression. Oncogene.

[55] Budanov A.V. (2014). The role of tumor suppressor p53 in the antioxidant defense and metabolism. Subcell. Biochem..

[56] Burkhart D.L., Sage J. (2008). Cellular Mechanisms of Tumour Suppression by the Retinoblastoma Gene. Nat. Rev. Cancer.

[57] Shi D., Gu W. (2012). Dual Roles of MDM2 in the Regulation of p53: Ubiquitination Dependent and Ubiquitination Independent Mechanisms of MDM2 Repression of p53 Activity. Genes Cancer.

[58] Pistritto G., Trisciuoglio D., Ceci C., Garufi A., D’Orazi G. (2016). Apoptosis as Anticancer Mechanism: Function and Dysfunction of its Modulators and Targeted Therapeutic Strategies. Aging.

[59] Roché H., Vahdat L.T. (2011). Treatment of Metastatic Breast Cancer: Second Line and Beyond. Ann. Oncol..

[60] Cummings M. (1996). Apoptosis of Epithelial Cells in Vivo Involves Tissue Transglutaminase Upregulation. J. Pathol..

[61] Piacentini M., D’Eletto M., Falasca L., Farrace M.G., Rodolfo C. (2011). Transglutaminase 2 at the Crossroads between Cell Death and Survival. Adv. Enzymol. Relat. Subj..

[62] Fok J.Y., Mehta K. (2007). Tissue Transglutaminase Induces the Release of Apoptosis Inducing Factor and Results in Apoptotic Death of Pancreatic Cancer Cells. Apoptosis.

[63] Oliverio S., Amendola A., Rodolfo C., Spinedi A., Piacentini M. (1999). Inhibition of “Tissue” Transglutaminase Increases Cell Survival by Preventing Apoptosis. J. Biol. Chem..

[64] Tatsukawa H., Fukaya Y., Frampton G., Martinez–Fuentes A., Suzuki K., Kuo T., Nagatsuma K., Shimokado K., Okuno M., Wu J. (2009). Role of Transglutaminase 2 in Liver Injury Via Cross-Linking and Silencing of Transcription Factor Sp1. Gastroenterology.

[65] Antonyak M.A., Jansen J.M., Miller A.M., Ly T.K., Endo M., Cerione R.A. (2006). Two Isoforms of Tissue Transglutaminase Mediate Opposing Cellular Fates. Proc. Natl. Acad. Sci. USA.

[66] Milakovic T., Tucholski J., McCoy E., Johnson G.V.W. (2004). Intracellular Localization and Activity State of Tissue Transglutaminase Differentially Impacts Cell Death. J. Biol. Chem..

[67] Fabbi M., Marimpietri D., Martini S., Brancolini C., Amoresano A., Scaloni A., Bargellesi A., Cosulich E. (1999). Tissue Transglutaminase is a Caspase Substrate during Apoptosis. Cleavage Causes Loss of Transamidating Function and is a Biochemical Marker of Caspase 3 Activation. Cell Death Differ..

[68] Lai T., Liu Y., Li W., Greenberg C.S. (2007). Identification of two GTP-independent alternatively spliced forms of tissue transglutaminase in human leukocytes, vascular smooth muscle, and endothelial cells. FASEB J..

[69] Eligini S., Fiorelli S., Tremoli E., Colli S. (2016). Inhibition of Transglutaminase 2 Reduces Efferocytosis in Human Macrophages: Role of CD14 and SR-AI Receptors. Nutr. Metab. Cardiovasc. Dis..

[70] Yen J., Yang D., Chen M., Yi-Ying W., Hsieh Y., Cheng Y., Huang W., Szondy Z., Tsay G.J. (2015). Corrigendum to “Daidzein Enhances Efferocytosis Via Transglutaminase 2 and Augmentation of Rac1 Activity” [*Mol. Immunol.*
**2014**, *60*, 135–142]. Mol. Immunol..

[71] Nicholas B., Smethurst P., Verderio E., Jones R., Griffin M. (2003). Cross-Linking of Cellular Proteins by Tissue Transglutaminase during Necrotic Cell Death: A Mechanism for Maintaining Tissue Integrity. Biochem. J..

[72] Abdulghani J., El-Deiry W.S. (2010). TRAIL Receptor Signaling and Therapeutics. Expert Opin. Ther. Targets.

[73] Mehta K. (1994). High Levels of Transglutaminase Expression in Doxorubicin-resistant Human Breast Carcinoma Cells. Int. J. Cancer.

[74] Boroughs L.K., Antonyak M.A., Cerione R.A. (2014). A novel Mechanism by which Tissue Transglutaminase Activates Signaling Events that Promote Cell Survival. J. Biol. Chem..

[75] Cao J., Huang W. (2016). Compensatory Increase of Transglutaminase 2 is Responsible for Resistance to mTOR Inhibitor Treatment. PLoS ONE.

[76] Carbone C., Carbone C., Di Gennaro E., di Gennaro E., Piro G., Piro G., Milone M., Milone M., Pucci B., Pucci B. (2017). Tissue Transglutaminase (TG2) is Involved in the Resistance of Cancer Cells to the Histone Deacetylase (HDAC) Inhibitor Vorinostat. Amino Acids.

[77] Glick D., Barth S., Macleod K.F. (2010). Autophagy: Cellular and Molecular Mechanisms. J. Pathol..

[78] Yun C., Lee S. (2018). The Roles of Autophagy in Cancer. Int. J. Mol. Sci..

[79] Jung H.J., Chen Z., Wang M., Fayad L., Romaguera J., Kwak L.W., McCarty N. (2012). Calcium Blockers Decrease the Bortezomib Resistance in Mantle Cell Lymphoma Via Manipulation of Tissue Transglutaminase Activities. Blood.

[80] Zhang H., Chen Z., Miranda R.N., Medeiros L.J., McCarty N. (2016). TG2 and NF-κB Signaling Coordinates the Survival of Mantle Cell Lymphoma Cells Via IL6-Mediated Autophagy. Cancer Res..

[81] Luciani A., Villella V.R., Esposito S., Brunetti-Pierri N., Medina D., Settembre C., Gavina M., Pulze L., Giardino I., Pettoello-Mantovani M. (2010). Defective CFTR Induces Aggresome Formation and Lung Inflammation in Cystic Fibrosis through ROS-Mediated Autophagy Inhibition. Nat. Cell Biol..

[82] Chaudhari N., Talwar P., Parimisetty A., d’Hellencourt L.C., Ravanan P. (2014). A Molecular Web: Endoplasmic Reticulum Stress, Inflammation, and Oxidative Stress. Front. Cell. Neurosci..

[83] Prasad S., Gupta S.C., Tyagi A.K. (2017). Reactive Oxygen Species (ROS) and Cancer: Role of Antioxidative Nutraceuticals. Cancer Lett..

[84] Hayflick L., Moorhead P.S. (1961). The Serial Cultivation of Human Diploid Cell Strains. Exp. Cell Res..

[85] Gilmore A.P. (2005). Anoikis. Cell Death Differ..

[86] Frisch S.M., Schaller M., Cieply B. (2013). Mechanisms that Link the Oncogenic Epithelial-Mesenchymal Transition to Suppression of Anoikis. J. Cell Sci..

[87] Verderio E.A.M., Telci D., Okoye A., Melino G., Griffin M. (2003). A Novel RGD-Independent Cell Adhesion Pathway Mediated by Fibronectin-Bound Tissue Transglutaminase Rescues Cells from Anoikis. J. Biol. Chem..

[88] Thapa R., Wilson G.D. (2016). The Importance of CD44 as a Stem Cell Biomarker and Therapeutic Target in Cancer. Stem Cells Int..

[89] Chen C., Zhao S., Karnad A., Freeman J.W. (2018). The Biology and Role of CD44 in Cancer Progression: Therapeutic Implications. J. Hematol. Oncol..

[90] Oh K., Lee O., Park Y., Seo M.W., Lee D. (2016). IL-1β Induces IL-6 Production and Increases Invasiveness and Estrogen-Independent Growth in a TG2-Dependent Manner in Human Breast Cancer Cells. BMC Cancer.

[91] Condello S., Sima L., Ivan C., Cardenas H., Schiltz G., Mishra R.K., Matei D. (2018). Tissue Tranglutaminase Regulates Interactions between Ovarian Cancer Stem Cells and the Tumor Niche. Cancer Res..

[92] Zhou Y., Bian S., Zhou X., Cui Y., Wang W., Wen L., Guo L., Fu W., Tang F. (2020). Single-Cell Multiomics Sequencing Reveals Prevalent Genomic Alterations in Tumor Stromal Cells of Human Colorectal Cancer. Cancer Cell.

[93] Eng C., Leone G., Orloff M.S., Ostrowski M.C. (2009). Genomic Alterations in Tumor Stroma. Cancer Res..

[94] Jakubek Y.A., Chang K., Sivakumar S., Yu Y., Giordano M.R., Fowler J., Huff C.D., Kadara H., Vilar E., Scheet P. (2020). Large-Scale Analysis of Acquired Chromosomal Alterations in Non-Tumor Samples from Patients with Cancer. Nat. Biotechnol..

[95] Joyce J.A., Pollard J.W. (2009). Microenvironmental Regulation of Metastasis. Nat. Rev. Cancer.

[96] Delaine-Smith R., Wright N., Hanley C., Hanwell R., Bhome R., Bullock M., Drifka C., Eliceiri K., Thomas G., Knight M. (2019). Transglutaminase-2 Mediates the Biomechanical Properties of the Colorectal Cancer Tissue Microenvironment that Contribute to Disease Progression. Cancers.

[97] Assi J., Srivastava G., Matta A., Chang M.C., Walfish P.G., Ralhan R. (2013). Transglutaminase 2 Overexpression in Tumor Stroma Identifies Invasive Ductal Carcinomas of Breast at High Risk of Recurrence. PLoS ONE.

[98] Fernández-Aceñero M.J., Torres S., Garcia-Palmero I., Díaz Del Arco C., Casal J.I. (2016). Prognostic role of tissue transglutaminase 2 in colon carcinoma. Virchows Arch..

[99] Wu J., Xu C., Guan X., Ni D., Yang X., Yang Z., Wang M. (2021). Comprehensive analysis of tumor microenvironment and identification of an immune signature to predict the prognosis and immunotherapeutic response in lung squamous cell carcinoma. Ann. Transl. Med..

[100] Stephens P., Grenard P., Aeschlimann P., Langley M., Blain E., Errington R., Kipling D., Thomas D., Aeschlimann D. (2004). Crosslinking and G-Protein Functions of Transglutaminase 2 Contribute Differentially to Fibroblast Wound Healing Responses. J. Cell Sci..

[101] Upchurch H.F., Conway E., Patterson M.K., Maxwell M.D. (1991). Localization of cellular transglutaminase on the extracellular matrix after wounding: Characteristics of the matrix bound enzyme. J. Cell. Physiol..

[102] Haroon Z.A., Hettasch J.M., Lai T.S., Dewhirst M.W., Greenberg C.S. (1999). Tissue transglutaminase is expressed, active, and directly involved in rat dermal wound healing and angiogenesis. FASEB J..

[103] Fell S., Wang Z., Blanchard A., Nanthakumar C., Griffin M. (2021). Transglutaminase 2: A novel therapeutic target for idiopathic pulmonary fibrosis using selective small molecule inhibitors. Amino Acids.

[104] Torres S., Garcia-Palmero I., Herrera M., Bartolomé R.A., Peña C., Fernandez-Aceñero M.J., Padilla G., Peláez-García A., Lopez-Lucendo M., Rodriguez-Merlo R. (2015). LOXL2 is Highly Expressed in Cancer-Associated Fibroblasts and Associates to Poor Colon Cancer Survival. Clin. Cancer Res..

[105] Hanley C.J., Waise S., Parker R., Lopez M.A., Taylor J., Kimbley L.M., West J., Ottensmeier C.H., Rose-Zerilli M.J.J., Thomas G.J. (2020). Spatially Discrete Signalling Niches Regulate Fibroblast Heterogeneity in Human Lung Cancer. bioRxiv.

[106] Elyada E., Bolisetty M., Laise P., Flynn W.F., Courtois E.T., Burkhart R.A., Teinor J.A., Belleau P., Biffi G., Lucito M.S. (2019). Cross-Species Single-Cell Analysis of Pancreatic Ductal Adenocarcinoma Reveals Antigen-Presenting Cancer-Associated Fibroblasts. Cancer Discov..

[107] Öhlund D., Handly-Santana A., Biffi G., Elyada E., Almeida A.S., Ponz-Sarvise M., Corbo V., Oni T.E., Hearn S.A., Lee E.J. (2017). Distinct Populations of Inflammatory Fibroblasts and Myofibroblasts in Pancreatic Cancer. J. Exp. Med..

[108] Mellone M., Hanley C.J., Thirdborough S., Mellows T., Garcia E., Woo J., Tod J., Frampton S., Jenei V., Moutasim K.A. (2016). Induction of Fibroblast Senescence Generates a Non-Fibrogenic Myofibroblast Phenotype that Differentially Impacts on Cancer Prognosis. Aging.

[109] Hariton F., Xue M., Rabbani N., Fowler M., Thornalley P.J. (2018). Sulforaphane Delays Fibroblast Senescence by Curbing Cellular Glucose Uptake, Increased Glycolysis, and Oxidative Damage. Oxidative Med. Cell. Longev..

[110] Sahai E., Astsaturov I., Cukierman E., DeNardo D.G., Egeblad M., Evans R.M., Fearon D., Greten F.R., Hingorani S.R., Hunter T. (2020). A Framework for Advancing our Understanding of Cancer-Associated Fibroblasts. Nat. Rev. Cancer.

[111] Lewis M.P., Lygoe K.A., Nystrom M.L., Anderson W.P., Speight P.M., Marshall J.F., Thomas G.J. (2004). Tumour-Derived TGF-Beta1 Modulates Myofibroblast Differentiation and Promotes HGF/SF-Dependent Invasion of Squamous Carcinoma Cells. Br. J. Cancer.

[112] Zeisberg E.M., Tarnavski O., Zeisberg M., Dorfman A.L., McMullen J.R., Gustafsson E., Chandraker A., Yuan X., Pu W.T., Roberts A.B. (2007). Endothelial-to-Mesenchymal Transition Contributes to Cardiac Fibrosis. Nat. Med..

[113] Cirri P., Chiarugi P. (2011). Cancer Associated Fibroblasts: The Dark Side of the Coin. Am. J. Cancer Res..

[114] Grauel A.L., Nguyen B., Ruddy D., Laszewski T., Schwartz S., Chang J., Chen J., Piquet M., Pelletier M., Yan Z. (2020). TGFβ-Blockade Uncovers Stromal Plasticity in Tumors by Revealing the Existence of a Subset of Interferon-Licensed Fibroblasts. Nat. Commun..

[115] George M.D., Vollberg T.M., Floyd E.E., Stein J.P., Jetten A.M. (1990). Regulation of Transglutaminase Type II by Transforming Growth Factor-Beta 1 in Normal and Transformed Human Epidermal Keratinocytes. J. Biol. Chem..

[116] Verderio E., Gaudry C., Gross S., Smith C., Downes S., Griffin M. (1999). Regulation of Cell Surface Tissue Transglutaminase: Effects on Matrix Storage of Latent Transforming Growth Factor-Β Binding Protein-1. J. Histochem. Cytochem..

[117] Nunes I., Gleizes P., Metz C.N., Rifkin D.B. (1997). Latent Transforming Growth Factor-Β Binding Protein Domains Involved in Activation and Transglutaminase-Dependent Cross-Linking of Latent Transforming Growth Factor-B. J. Cell Biol..

[118] Jia C., Wang G., Wang T., Fu B., Zhang Y., Huang L., Deng Y., Chen G., Wu X., Chen J. (2020). Cancer-Associated Fibroblasts Induce Epithelial-Mesenchymal Transition Via the Transglutaminase 2-Dependent IL-6/IL6R/STAT3 Axis in Hepatocellular Carcinoma. Int. J. Biol. Sci..

[119] Saghy T., Koroskenyi K., Hegedus K., Antal M., Banko C., Bacso Z., Papp A., Stienstra R., Szondy Z. (2019). Loss of Transglutaminase 2 Sensitizes for Diet-Induced Obesity-Related Inflammation and Insulin Resistance due to Enhanced Macrophage C-Src Signaling. Cell Death Dis..

[120] Penumatsa K.C., Falcão-Pires I., Leite S., Leite-Moreira A., Bhedi C.D., Nasirova S., Ma J., Sutliff R.L., Fanburg B.L. (2020). Increased Transglutaminase 2 Expression and Activity in Rodent Models of Obesity/Metabolic Syndrome and Aging. Front. Physiol..

[121] Zhao C., Wu M., Zeng N., Xiong M., Hu W., Lv W., Yi Y., Zhang Q., Wu Y. (2020). Cancer-Associated Adipocytes: Emerging Supporters in Breast Cancer. J. Exp. Clin. Cancer Res..

[122] Cai Z., Liang Y., Xing C., Wang H., Hu P., Li J., Huang H., Wang W., Jiang C. (2019). Cancer-associated Adipocytes Exhibit Distinct Phenotypes and Facilitate Tumor Progression in Pancreatic Cancer. Oncol. Rep..

[123] Wen Y., Xing X., Harris J.W., Zaytseva Y.Y., Mitov M.I., Napier D.L., Weiss H.L., Mark Evers B., Gao T. (2017). Adipocytes Activate Mitochondrial Fatty Acid Oxidation and Autophagy to Promote Tumor Growth in Colon Cancer. Cell Death Dis..

[124] Bochet L., Lehuede C., Dauvillier S., Wang Y.Y., Dirat B., Laurent V., Dray C., Guiet R., Maridonneau-Parini I., Le Gonidec S. (2013). Adipocyte-Derived Fibroblasts Promote Tumor Progression and Contribute to the Desmoplastic Reaction in Breast Cancer. Cancer Res..

[125] Myneni V.D., Melino G., Kaartinen M.T. (2015). Transglutaminase 2-a Novel Inhibitor of Adipogenesis. Cell Death Dis..

[126] Maiuri L., Luciani A., Giardino I., Raia V., Villella V.R., D’Apolito M., Pettoello-Mantovani M., Guido S., Ciacci C., Cimmino M. (2008). Tissue Transglutaminase Activation Modulates Inflammation in Cystic Fibrosis Via PPARgamma Down-Regulation. J. Immunol..

[127] Klöck C., DiRaimondo T., Khosla C. (2012). Role of Transglutaminase 2 in Celiac Disease Pathogenesis. Semin. Immunopathol..

[128] Johnson T.S., Fisher M., Haylor J.L., Hau Z., Skill N.J., Jones R., Saint R., Coutts I., Vickers M.E., El Nahas A.M. (2007). Transglutaminase Inhibition Reduces Fibrosis and Preserves Function in Experimental Chronic Kidney Disease. J. Am. Soc. Nephrol..

[129] Su T., Qin X., Furutani Y. (2021). Transglutaminase 2 as a Marker for Inflammation and Therapeutic Target in Sepsis. Int. J. Mol. Sci..

[130] Bijli K., Kanter B., Minhajuddin M., Leonard A., Xu L., Fazal F., Rahman A. (2014). Regulation of Endothelial Cell Inflammation and Lung Polymorphonuclear Lymphocyte Infiltration by Transglutaminase 2. Shock.

[131] Lee J., Kim Y., Choi D., Bang M.S., Han T.R., Joh T.H., Kim S. (2004). Transglutaminase 2 Induces Nuclear Factor-kappaB Activation Via a Novel Pathway in BV-2 Microglia. J. Biol. Chem..

[132] Mirza A., Liu S.L., Frizell E., Zhu J., Maddukuri S., Martinez J., Davies P., Schwarting R., Norton P., Zern M.A. (1997). A Role for Tissue Transglutaminase in Hepatic Injury and Fibrogenesis, and its Regulation by NF-kappaB. Am. J. Physiol. Gastrointest. Liver Physiol..

[133] Masjedi A., Hashemi V., Hojjat-Farsangi M., Ghalamfarsa G., Azizi G., Yousefi M., Jadidi-Niaragh F. (2018). The Significant Role of Interleukin-6 and its Signaling Pathway in the Immunopathogenesis and Treatment of Breast Cancer. Biomed. Pharmacother..

[134] Oh K., Moon H., Lee D., Yoo Y. (2015). Tissue Transglutaminase-Interleukin-6 Axis Facilitates Peritoneal Tumor Spreading and Metastasis of Human Ovarian Cancer Cells. Lab. Anim. Res..

[135] Bharti R., Dey G., Mandal M. (2016). Cancer Development, Chemoresistance, Epithelial to Mesenchymal Transition and Stem Cells: A Snapshot of IL-6 Mediated Involvement. Cancer Lett..

[136] Gonzalez H., Hagerling C., Werb Z. (2018). Roles of the Immune System in Cancer: From Tumor Initiation to Metastatic Progression. Genes Dev..

[137] Nielsen S.R., Schmid M.C. (2017). Macrophages as Key Drivers of Cancer Progression and Metastasis. Mediat. Inflamm..

[138] Van Strien M.E., de Vries H.E., Chrobok N.L., Bol J.G.J.M., Breve J.J.P., van der Pol S.M.P., Kooij G., van Buul J.D., Karpuj M., Steinman L. (2015). Tissue Transglutaminase Contributes to Experimental Multiple Sclerosis Pathogenesis and Clinical Outcome by Promoting Macrophage Migration. Brain Behav. Immun..

[139] Najafi M., Hashemi Goradel N., Farhood B., Salehi E., Nashtaei M.S., Khanlarkhani N., Khezri Z., Majidpoor J., Abouzaripour M., Habibi M. (2019). Macrophage Polarity in Cancer: A Review. J. Cell. Biochem..

[140] Sun H., Kaartinen M.T. (2018). Transglutaminases in Monocytes and Macrophages. Med. Sci..

[141] Sestito C., Brevé J.J.P., Bol J.G.J.M., Wilhelmus M.M.M., Drukarch B., van Dam A. (2020). Tissue Transglutaminase Contributes to Myelin Phagocytosis in Interleukin-4-Treated Human Monocyte-Derived Macrophages. Cytokine.

[142] Nadella V., Wang Z., Johnson T.S., Griffin M., Devitt A. (2015). Transglutaminase 2 Interacts with Syndecan-4 and CD44 at the Surface of Human Macrophages to Promote Removal of Apoptotic Cells. Biochim. Biophys. Acta Mol. Cell Res..

[143] Scarpellini A., Huang L., Burhan I., Schroeder N., Funck M., Johnson T.S., Verderio E.A.M. (2014). Syndecan-4 Knockout Leads to Reduced Extracellular Transglutaminase-2 and Protects Against Tubulointerstitial Fibrosis. J. Am. Soc. Nephrol..

[144] Benn M.C., Weber W., Klotzsch E., Vogel V., Pot S.A. (2019). Tissue Transglutaminase in Fibrosis—More than an Extracellular Matrix Cross-Linker. Curr. Opin. Biomed. Eng..

[145] Afik R., Zigmond E., Vugman M., Klepfish M., Shimshoni E., Pasmanik-Chor M., Shenoy A., Bassat E., Halpern Z., Geiger T. (2016). Tumor Macrophages are Pivotal Constructors of Tumor Collagenous Matrix. J. Exp. Med..

[146] Liguori M., Solinas G., Germano G., Mantovani A., Allavena P. (2011). Tumor-Associated Macrophages as Incessant Builders and Destroyers of the Cancer Stroma. Cancers.

[147] Quaranta V., Rainer C., Nielsen S.R., Raymant M.L., Ahmed M.S., Engle D.D., Taylor A., Murray T., Campbell F., Palmer D.H. (2018). Macrophage-Derived Granulin Drives Resistance to Immune Checkpoint Inhibition in Metastatic Pancreatic Cancer. Cancer Res..

[148] Hodrea J., Demény M.Á., Majai G., Sarang Z., Korponay-Szabó I.R., Fésüs L. (2010). Transglutaminase 2 is Expressed and Active on the Surface of Human Monocyte-Derived Dendritic Cells and Macrophages. Immunol. Lett..

[149] Kim J., Jeong E.M., Jeong Y., Lee W.J., Kang J.S., Kim I., Hwang Y. (2014). Transglutaminase 2 on the Surface of Dendritic Cells is Proposed to be Involved in Dendritic cell–T Cell Interaction. Cell. Immunol..

[150] Ráki M., Schjetne K.W., Stamnaes J., Molberg Ø., Jahnsen F.L., Issekutz T.B., Bogen B., Sollid L.M. (2007). Surface Expression of Transglutaminase 2 by Dendritic Cells and its Potential Role for Uptake and Presentation of Gluten Peptides to T Cells. Scand. J. Immunol..

[151] Ghoneim H.E., Zamora A.E., Thomas P.G., Youngblood B.A. (2016). Cell-Intrinsic Barriers of T Cell-Based Immunotherapy. Trends Mol. Med..

[152] Ando M., Ito M., Srirat T., Kondo T., Yoshimura A. (2020). Memory T Cell, Exhaustion, and Tumor Immunity. Immunol. Med..

[153] Liu J., Liu Q., Zhang X., Cui M., Li T., Zhang Y., Liao Q. (2021). Immune Subtyping for Pancreatic Cancer with Implication in Clinical Outcomes and Improving Immunotherapy. Cancer Cell Int..

[154] Yadav L. (2015). Tumour Angiogenesis and Angiogenic Inhibitors: A Review. J. Clin. Diagn. Res..

[155] Lunt S.Y., Vander Heiden M.G. (2011). Aerobic Glycolysis: Meeting the Metabolic Requirements of Cell Proliferation. Annu. Rev. Cell Dev. Biol..

[156] Schwartz L., Supuran C.T., Alfarouk O.K. (2017). The Warburg Effect and the Hallmarks of Cancer. Anti-Cancer Agents Med. Chem..

[157] Mastroberardino P.G., Farrace M.G., Viti I., Pavone F., Fimia G.M., Melino G., Rodolfo C., Piacentini M. (2006). “Tissue” transglutaminase contributes to the formation of disulphide bridges in proteins of mitochondrial respiratory complexes. Biochim. Biophys. Acta.

[158] Rossin F., D’Eletto M., Falasca L., Sepe S., Cocco S., Fimia G.M., Campanella M., Mastroberardino P.G., Farrace M.G., Piacentini M. (2015). Transglutaminase 2 ablation leads to mitophagy impairment associated with a metabolic shift towards aerobic glycolysis. Cell Death Differ..

[159] Bhedi C.D., Nasirova S., Toksoz D., Warburton R.R., Morine K.J., Kapur N.K., Galper J.B., Preston I.R., Hill N.S., Fanburg B.L. (2020). Glycolysis regulated transglutaminase 2 activation in cardiopulmonary fibrogenic remodeling. FASEB J..

[160] Katt W.P., Antonyak M.A., Cerione R.A. (2015). Simultaneously Targeting Tissue Transglutaminase and Kidney Type Glutaminase Sensitizes Cancer Cells to Acid Toxicity and Offers New Opportunities for Therapeutic Intervention. Mol. Pharm..

[161] Brooks S.A., Lomax-Browne H.J., Carter T.M., Kinch C.E., Hall D.M.S. (2010). Molecular Interactions in Cancer Cell Metastasis. Acta Histochem..

[162] Greijer A.E., van der Wall E. (2004). The Role of Hypoxia Inducible Factor 1 (HIF-1) in Hypoxia Induced Apoptosis. J. Clin. Pathol..

[163] Lossi L., Castagna C., Merighi A. (2018). Caspase-3 Mediated Cell Death in the Normal Development of the Mammalian Cerebellum. Int. J. Mol. Sci..

[164] Baeriswyl V., Christofori G. (2009). The Angiogenic Switch in Carcinogenesis. Semin. Cancer Biol..

[165] Carmeliet P., Jain R.K. (2011). Molecular Mechanisms and Clinical Applications of Angiogenesis. Nature.

[166] Cooke V., LeBleu V., Keskin D., Khan Z., O’Connell J., Teng Y., Duncan M., Xie L., Maeda G., Vong S. (2012). Pericyte Depletion Results in Hypoxia-Associated Epithelial-to-Mesenchymal Transition and Metastasis Mediated by Met Signaling Pathway. Cancer Cell.

[167] Jones R.A., Kotsakis P., Johnson T.S., Chau D.Y.S., Ali S., Melino G., Griffin M. (2006). Matrix Changes Induced by Transglutaminase 2 Lead to Inhibition of Angiogenesis and Tumor Growth. Cell Death Differ..

[168] Stylianopoulos T., Martin J.D., Chauhan V.P., Jain S.R., Diop-Frimpong B., Bardeesy N., Smith B.L., Ferrone C.R., Hornicek F.J., Boucher Y. (2012). Causes, Consequences, and Remedies for Growth-Induced Solid Stress in Murine and Human Tumors. Proc. Natl. Acad. Sci. USA.

[169] Spurlin T.A., Bhadriraju K., Chung K., Tona A., Plant A.L. (2009). Vascular Smooth Muscle Cell Response to Transglutaminase 2 Cross-Linked Collagen Fibril Thin Films. Biophys. J..

[170] Chauhan V.P., Martin J.D., Liu H., Lacorre D.A., Jain S.R., Kozin S.V., Stylianopoulos T., Mousa A.S., Han X., Adstamongkonkul P. (2013). Angiotensin Inhibition Enhances Drug Delivery and Potentiates Chemotherapy by Decompressing Tumor Blood Vessels. Nat. Commun..

[171] Chauhan V.P., Chen I.X., Tong R., Ng M.R., Martin J.D., Naxerova K., Wu M.W., Huang P., Boucher Y., Kohane D.S. (2019). Reprogramming the Microenvironment with Tumor-Selective Angiotensin Blockers Enhances Cancer Immunotherapy. Proc. Natl. Acad. Sci. USA.

[172] Paszek M.J., Zahir N., Johnson K.R., Lakins J.N., Rozenberg G.I., Gefen A., Reinhart-King C.A., Margulies S.S., Dembo M., Boettiger D. (2005). Tensional Homeostasis and the Malignant Phenotype. Cancer Cell.

[173] Pickup M.W., Mouw J.K., Weaver V.M. (2014). The Extracellular Matrix Modulates the Hallmarks of Cancer. EMBO Rep..

[174] Yue B. (2014). Biology of the Extracellular Matrix: An Overview. J. Glaucoma.

[175] Nia H.T., Munn L.L., Jain R.K. (2020). Physical Traits of Cancer. Science.

[176] Jain R.K., Martin J.D., Stylianopoulos T. (2014). The Role of Mechanical Forces in Tumor Growth and Therapy. Annu. Rev. Biomed. Eng..

[177] Kumar S., Weaver V. (2009). Mechanics, Malignancy, and Metastasis: The Force Journey of a Tumor Cell. Cancer Metastasis Rev..

[178] Kalli M., Papageorgis P., Gkretsi V., Stylianopoulos T. (2018). Solid Stress Facilitates Fibroblasts Activation to Promote Pancreatic Cancer Cell Migration. Ann. Biomed. Eng..

[179] Wipff P., Rifkin D.B., Meister J., Hinz B. (2007). Myofibroblast Contraction Activates Latent TGF-Β1 from the Extracellular Matrix. J. Cell Biol..

[180] Semkova M.E., Hsuan J.J. (2021). TGFβ-1 Induced Cross-Linking of the Extracellular Matrix of Primary Human Dermal Fibroblasts. Int. J. Mol. Sci..

[181] Zhang R., Ma M., Dong G., Yao R., Li J., Zheng Q., Dong Y., Ma H., Gao D., Cui J. (2017). Increased Matrix Stiffness Promotes Tumor Progression of Residual Hepatocellular Carcinoma After Insufficient Heat Treatment. Cancer Sci..

[182] Zhao D., Xue C., Li Q., Liu M., Ma W., Zhou T., Lin Y. (2018). Substrate Stiffness Regulated Migration and Angiogenesis Potential of A549 Cells and HUVECs. J. Cell. Physiol..

[183] Berger A.J., Renner C.M., Hale I., Yang X., Ponik S.M., Weisman P.S., Masters K.S., Kreeger P.K. (2020). Scaffold Stiffness Influences Breast Cancer Cell Invasion Via EGFR-Linked Mena Upregulation and Matrix Remodeling. Matrix Biol..

[184] Reid S.E., Kay E.J., Neilson L.J., Henze A., Serneels J., McGhee E.J., Dhayade S., Nixon C., Mackey J.B., Santi A. (2017). Tumor Matrix Stiffness Promotes Metastatic Cancer Cell Interaction with the Endothelium. EMBO J..

[185] Saatci O., Kaymak A., Raza U., Ersan P.G., Akbulut O., Banister C.E., Sikirzhytski V., Tokat U.M., Aykut G., Ansari S.A. (2020). Targeting Lysyl Oxidase (LOX) Overcomes Chemotherapy Resistance in Triple Negative Breast Cancer. Nat. Commun..

[186] Lee J., Condello S., Yakubov B., Emerson R., Caperell-Grant A., Hitomi K., Xie J., Matei D. (2015). Tissue Transglutaminase Mediated Tumor-Stroma Interaction Promotes Pancreatic Cancer Progression. Clinical Cancer Res..

[187] Mohammadi H., Sahai E. (2018). Mechanisms and Impact of Altered Tumour Mechanics. Nat. Cell Biol..

[188] Pankova D., Chen Y., Terajima M., Schliekelman M.J., Baird B.N., Fahrenholtz M., Sun L., Gill B.J., Vadakkan T.J., Kim M.P. (2016). Cancer-Associated Fibroblasts Induce a Collagen Cross-Link Switch in Tumor Stroma. Mol. Cancer Res..

[189] Wirtz D., Konstantopoulos K., Searson P.C. (2011). The Physics of Cancer: The Role of Physical Interactions and Mechanical Forces in Metastasis. Nat. Rev. Cancer.

[190] Friedl P.H.A., Gilmour D. (2009). Collective Cell Migration in Morphogenesis, Regeneration and Cancer. Nat. Rev. Mol. Cell Biol..

[191] Tse J.M., Cheng G., Tyrrell J.A., Wilcox-Adelman S.A., Boucher Y., Jain R.K., Munn L.L. (2011). Mechanical Compression Drives Cancer Cells Toward Invasive Phenotype. Proc. Natl. Acad. Sci. USA.

[192] Tsai J.H., Yang J. (2013). Epithelial-Mesenchymal Plasticity in Carcinoma Metastasis. Genes Dev..

[193] Mierke C.T. (2019). The Matrix Environmental and Cell Mechanical Properties Regulate Cell Migration and Contribute to the Invasive Phenotype of Cancer Cells. Rep. Prog. Phys..

[194] Chen S., Lin C., Lee M., Lee L., Chang G., Lee P., Hung C., Ko W., Tsai P., Schally A.V. (2010). Up-Regulation of Fibronectin and Tissue Transglutaminase Promotes Cell Invasion Involving Increased Association with Integrin and MMP Expression in A431 Cells. Anticancer Res..

[195] Bordeleau F., Wang W., Simmons A., Antonyak M.A., Cerione R.A., Reinhart-King C.A. (2020). Tissue Transglutaminase 2 Regulates Tumor Cell Tensional Homeostasis by Increasing Contractility. J. Cell Sci..

[196] Yurchenco P.D., Ruben G.C. (1987). Basement Membrane Structure in Situ: Evidence for Lateral Associations in the Type IV Collagen Network. J. Cell Biol..

[197] LeBleu V.S., MacDonald B., Kalluri R. (2007). Structure and Function of Basement Membranes. Exp. Biol. Med..

[198] Iozzo R.V., Zoeller J.J., Nyström A. (2009). Basement Membrane Proteoglycans: Modulators Par Excellence of Cancer Growth and Angiogenesis. Mol. Cells.

[199] Wisdom K.M., Indana D., Chou P., Desai R., Kim T., Chaudhuri O. (2020). Covalent Cross-Linking of Basement Membrane-Like Matrices Physically Restricts Invasive Protrusions in Breast Cancer Cells. Matrix Biol..

[200] Hotary K., Li X., Allen E., Stevens S.L., Weiss S.J. (2006). A Cancer Cell Metalloprotease Triad Regulates the Basement Membrane Transmigration Program. Genes Dev..

[201] Chang J., Chaudhuri O. (2019). Beyond Proteases: Basement Membrane Mechanics and Cancer Invasion. J. Cell Biol..

[202] Strzyz P. (2019). Forcing through Barriers. Nat. Rev. Mol. Cell Biol..

[203] Kalluri R. (2003). Basement Membranes: Structure, Assembly and Role in Tumour Angiogenesis. Nat. Rev. Cancer.

[204] Philp C.J., Siebeke I., Clements D., Miller S., Habgood A., John A.E., Navaratnam V., Hubbard R.B., Jenkins G., Johnson S.R. (2018). Extracellular Matrix Cross-Linking Enhances Fibroblast Growth and Protects Against Matrix Proteolysis in Lung Fibrosis. Am. J. Respir. Cell Mol. Biol..

[205] Aeschlimann D., Paulsson M. (1991). Cross-Linking of Laminin-Nidogen Complexes by Tissue Transglutaminase. A Novel Mechanism for Basement Membrane Stabilization. J. Biol. Chem..

[206] Dziadek M. (1995). Role of Laminin-Nidogen Complexes in Basement Membrane Formation during Embryonic Development. Experientia.

[207] Haroon Z.A., Lai T., Hettasch J.M., Lindberg R.A., Dewhirst M.W., Greenberg C.S. (1999). Tissue Transglutaminase is Expressed as a Host Response to Tumor Invasion and Inhibits Tumor Growth. Lab. Investig..

[208] Mangala L.S., Arun B., Sahin A.A., Mehta K. (2005). Tissue Transglutaminase-Induced Alterations in Extracellular Matrix Inhibit Tumor Invasion. Mol. Cancer.

[209] Di Giacomo G., Di Giacomo G., Lentini A., Lentini A., Beninati S., Beninati S., Piacentini M., Piacentini M., Rodolfo C., Rodolfo C. (2009). In Vivo Evaluation of Type 2 Transglutaminase Contribution to the Metastasis Formation in Melanoma. Amino Acids.

[210] Facchiano F., Facchiano F., D’Arcangelo D., D’Arcangelo D., Lentini A., Lentini A., Rossi S., Rossi S., Senatore C., Senatore C. (2013). Tissue Transglutaminase Activity Protects from Cutaneous Melanoma Metastatic Dissemination: An in Vivo Study. Amino Acids.

[211] Levental K.R., Yu H., Kass L., Lakins J.N., Egeblad M., Erler J.T., Fong S.F.T., Csiszar K., Giaccia A., Weninger W. (2009). Matrix Crosslinking Forces Tumor Progression by Enhancing Integrin Signaling. Cell.

[212] Satpathy M., Shao M., Emerson R., Donner D.B., Matei D. (2009). Tissue Transglutaminase Regulates Matrix Metalloproteinase-2 in Ovarian Cancer by Modulating cAMP-Response Element-Binding Protein Activity. J. Biol. Chem..

[213] Coulson-Thomas V., Coulson-Thomas Y., Gesteira T., de Paula C., Mader A., Waisberg J., Pinhal M., Friedl A., Toma L., Nader H. (2011). Colorectal Cancer Desmoplastic Reaction Up-Regulates Collagen Synthesis and Restricts Cancer Cell Invasion. Cell Tissue Res..

[214] Chau D.Y.S., Collighan R.J., Verderio E.A.M., Addy V.L., Griffin M. (2005). The Cellular Response to Transglutaminase-Cross-Linked Collagen. Biomaterials.

[215] Belkin A.M., Zemskov E.A., Hang J., Akimov S.S., Sikora S., Strongin A.Y. (2004). Cell-Surface-Associated Tissue Transglutaminase is a Target of MMP-2 Proteolysis. Biochemistry.

[216] Belkin A.M., Akimov S.S., Zaritskaya L.S., Ratnikov B.I., Deryugina E.I., Strongin A.Y. (2001). Matrix-Dependent Proteolysis of Surface Transglutaminase by Membrane-Type Metalloproteinase Regulates Cancer Cell Adhesion and Locomotion. J. Biol. Chem..

[217] Birckbichler P.J., Bonner R.B., Hurst R.E., Bane B.L., Pitha J.V., Hemstreet G.P. (2000). Loss of tissue transglutaminase as a biomarker for prostate adenocarcinoma. Cancer.

[218] Hager H., Jensen P.H., Hamilton-Dutoit S., Neilsen M.S., Birckbichler P., Gliemann J. (1997). Expression of tissue transglutaminase in human bladder carcinoma. J. Pathol..

[219] Baghban R., Roshangar L., Jahanban-Esfahlan R., Seidi K., Ebrahimi-Kalan A., Jaymand M., Kolahian S., Javaheri T., Zare P. (2020). Tumor Microenvironment Complexity and Therapeutic Implications at a Glance. Cell Commun. Signal..

[220] Naba A., Clauser K.R., Ding H., Whittaker C.A., Carr S.A., Hynes R.O. (2016). The Extracellular Matrix: Tools and Insights for the “omics” Era. Matrix Biol..

[221] Campbell I.D., Humphries M.J. (2011). Integrin Structure, Activation, and Interactions. Cold Spring Harb. Perspect. Biol..

[222] Fullár A., Dudás J., Oláh L., Hollósi P., Papp Z., Sobel G., Karászi K., Paku S., Baghy K., Kovalszky I. (2015). Remodeling of Extracellular Matrix by Normal and Tumor-Associated Fibroblasts Promotes Cervical Cancer Progression. BMC Cancer.

[223] Deville S.S., Cordes N. (2019). The Extracellular, Cellular, and Nuclear Stiffness, a Trinity in the Cancer Resistome—A Review. Front. Oncol..

[224] Cox T.R., Erler J.T. (2011). Remodeling and Homeostasis of the Extracellular Matrix: Implications for Fibrotic Diseases and Cancer. Dis. Models Mech..

[225] Huijbers I.J., Iravani M., Popov S., Robertson D., Al-Sarraj S., Jones C., Isacke C.M. (2010). A Role for Fibrillar Collagen Deposition and the Collagen Internalization Receptor Endo180 in Glioma Invasion. PLoS ONE.

[226] Distler J.H.W., Györfi A., Ramanujam M., Whitfield M.L., Königshoff M., Lafyatis R. (2019). Shared and Distinct Mechanisms of Fibrosis. Nat. Rev. Rheumatol..

[227] Hinz B., Phan S.H., Thannickal V.J., Prunotto M., Desmouliere A., Varga J., de Wever O., Mareel M., Gabbiani G. (2012). Recent Developments in Myofibroblast Biology: Paradigms for Connective Tissue Remodeling. Am. J. Pathol..

[228] Tschumperlin D.J., Lagares D. (2020). Mechano-Therapeutics: Targeting Mechanical Signaling in Fibrosis and Tumor Stroma. Pharmacol. Ther..

[229] Kauppila S., Stenbäck F., Risteli J., Jukkola A., Risteli L. (1998). Aberrant Type I and Type III Collagen Gene Expression in Human Breast Cancer in Vivo. J. Pathol..

[230] Bonnans C., Chou J., Werb Z. (2014). Remodelling the Extracellular Matrix in Development and Disease. Nat. Rev. Mol. Cell Biol..

[231] Wells R.G., Discher D.E. (2008). Matrix Elasticity, Cytoskeletal Tension, and TGF-Beta: The Insoluble and Soluble Meet. Sci. Signal..

[232] Mickle M., Adhikary G., Shrestha S., Xu W., Eckert R.L. (2021). VGLL4 inhibits YAP1/TEAD signaling to suppress the epidermal squamous cell carcinoma cancer phenotype. Mol. Carcinog..

[233] Herman J.F., Mangala L.S., Mehta K. (2006). Implications of Increased Tissue Transglutaminase (TG2) Expression in Drug-Resistant Breast Cancer (MCF-7) Cells. Oncogene.

[234] Akimov S.S., Krylov D., Fleischman L.F., Belkin A.M. (2000). Tissue Transglutaminase is an Integrin-Binding Adhesion Coreceptor for Fibronectin. J. Cell Biol..

[235] Kotsakis P., Kotsakis P., Wang Z., Wang Z., Collighan R., Collighan R., Griffin M., Griffin M. (2011). The Role of Tissue Transglutaminase (TG2) in Regulating the Tumour Progression of the Mouse Colon Carcinoma CT26. Amino Acids.

[236] Libring S., Shinde A., Chanda M.K., Nuru M., George H., Saleh A.M., Abdullah A., Kinzer-Ursem T.L., Calve S., Wendt M.K. (2020). The Dynamic Relationship of Breast Cancer Cells and Fibroblasts in Fibronectin Accumulation at Primary and Metastatic Tumor Sites. Cancers.

[237] Khanna M., Chelladurai B., Gavini A., Li L., Shao M., Courtney D., Turchi J.J., Matei D., Meroueh S. (2011). Targeting Ovarian Tumor Cell Adhesion Mediated by Tissue Transglutaminase. Mol. Cancer Ther..

[238] Mangala L.S., Fok J.Y., Zorrilla-Calancha I.R., Verma A., Mehta K. (2007). Tissue Transglutaminase Expression Promotes Cell Attachment, Invasion and Survival in Breast Cancer Cells. Oncogene.

[239] Parri M., Chiarugi P. (2010). Rac and Rho GTPases in Cancer Cell Motility Control. Cell Commun. Signal..

[240] Dillekås H., Rogers M.S., Straume O. (2019). Are 90% of Deaths from Cancer Caused by Metastases?. Cancer Med..

[241] Erdem S., Erdem S., Yegen G., Yegen G., Telci D., Telci D., Yildiz I., Yildiz I., Tefik T., Tefik T. (2015). The Increased Transglutaminase 2 Expression Levels during Initial Tumorigenesis Predict Increased Risk of Metastasis and Decreased Disease-Free and Cancer-Specific Survivals in Renal Cell Carcinoma. World J. Urol..

[242] Erdem M., Erdem S., Sanli O., Sak H., Kilicaslan I., Sahin F., Telci D. (2014). Up-Regulation of TGM2 with ITGB1 and SDC4 is Important in the Development and Metastasis of Renal Cell Carcinoma. Urol. Oncol..

[243] Seo S., Moon Y., Choi J., Yoon S., Jung K.H., Cheon J., Kim W., Kim D., Lee C.H., Kim S. (2019). The GTP Binding Activity of Transglutaminase 2 Promotes Bone Metastasis of Breast Cancer Cells by Downregulating microRNA-205. Am. J. Cancer Res..

[244] Fok J.Y., Ekmekcioglu S., Mehta K. (2006). Implications of Tissue Transglutaminase Expression in Malignant Melanoma. Mol. Cancer Ther..

[245] Antonyak M.A., Li B., Boroughs L.K., Johnson J.L., Druso J.E., Bryant K.L., Holowka D.A., Cerione R.A. (2011). Cancer Cell-Derived Microvesicles Induce Transformation by Transferring Tissue Transglutaminase and Fibronectin to Recipient Cells. Proc. Natl. Acad. Sci. USA.

[246] Van den Akker J., van Weert A., Afink G., Bakker E.N.T.P., van der Pol E., Böing A.N., Nieuwland R., VanBavel E. (2012). Transglutaminase 2 is Secreted from Smooth Muscle Cells by Transamidation-Dependent Microparticle Formation. Amino Acids.

[247] Bianchi N., Beninati S., Bergamini C.M. (2018). Spotlight on the Transglutaminase 2 Gene: A Focus on Genomic and Transcriptional Aspects. Biochem. J..

[248] Liu Y., Cao X. (2016). Characteristics and Significance of the Pre-Metastatic Niche. Cancer Cell.

[249] Hoshino A., Costa-Silva B., Shen T., Rodrigues G., Hashimoto A., Tesic Mark M., Molina H., Kohsaka S., Di Giannatale A., Ceder S. (2015). Tumour Exosome Integrins Determine Organotropic Metastasis. Nature.

[250] Shinde A., Paez J.S., Libring S., Hopkins K., Solorio L., Wendt M.K. (2020). Transglutaminase-2 Facilitates Extracellular Vesicle-Mediated Establishment of the Metastatic Niche. Oncogenesis.

[251] Shafiq A., Suwakulsiri W., Rai A., Chen M., Greening D.W., Zhu H.J., Xu R., Simpson R.J. (2021). Transglutaminase-2, RNA-binding proteins and mitochondrial proteins selectively traffic to MDCK cell-derived microvesicles following H-Ras-induced epithelial-mesenchymal transition. Proteomics.

[252] Brill-Karniely Y., Dror D., Duanis-Assaf T., Goldstein Y., Schwob O., Millo T., Orehov N., Stern T., Jaber M., Loyfer N. (2020). Triangular Correlation (TrC) between Cancer Aggressiveness, Cell Uptake Capability, and Cell Deformability. Sci. Adv..

[253] Eligula L., Chuang L., Phillips M.L., Motoki M., Seguro K., Muhlrad A. (1998). Transglutaminase-Induced Cross-Linking between Subdomain 2 of G-Actin and the 636–642 Lysine-Rich Loop of Myosin Subfragment 1. Biophys. J..

[254] Dolge L., Dolge L., Aufenvenne K., Aufenvenne K., Traupe H., Traupe H., Baumgartner W., Baumgartner W. (2012). Beta-Actin is a Target for Transglutaminase Activity at Synaptic Endings in Chicken Telencephalic Cell Cultures. J. Mol. Neurosci..

[255] Song Y., Kirkpatrick L., Schilling A., Helseth D., Chabot N., Keillor J., Johnson G.W., Brady S. (2013). Transglutaminase and Polyamination of Tubulin: Posttranslational Modification for Stabilizing Axonal Microtubules. Neuron.

[256] Schwager S.C., Bordeleau F., Zhang J., Antonyak M.A., Cerione R.A., Reinhart-King C.A. (2019). Matrix Stiffness Regulates Microvesicle-Induced Fibroblast Activation. Am. J. Physiol. Cell Physiol..

[257] Adamczyk M., Griffiths R., Dewitt S., Knäuper V., Aeschlimann D. (2015). P2X7 Receptor Activation Regulates Rapid Unconventional Export of Transglutaminase-2. J. Cell Sci..

[258] Arnaud-Sampaio V.F., Rabelo I.L.A., Ulrich H., Lameu C. (2020). The P2X7 Receptor in the Maintenance of Cancer Stem Cells, Chemoresistance and Metastasis. Stem Cell Rev. Rep..

[259] Wang-Gillam A., Lockhart A.C., Tan B.R., Suresh R., Lim K., Ratner L., DeNardo D.G. (2018). Phase I Study of Defactinib Combined with Pembrolizumab and Gemcitabine in Patients with Advanced Cancer. J. Clin. Oncol..

[260] Mohanty A., Pharaon R.R., Nam A., Salgia S., Kulkarni P., Massarelli E. (2020). FAK-Targeted and Combination Therapies for the Treatment of Cancer: An Overview of Phase I and II Clinical Trials. Expert Opin. Investig. Drugs.

[261] Akhurst R.J. (2017). Targeting TGF-Beta Signaling for Therapeutic Gain. Cold Spring Harb. Perspect. Biol..

[262] Raab-Westphal S., Marshall J.F., Goodman S.L. (2017). Integrins as Therapeutic Targets: Successes and Cancers. Cancers.

[263] Efthymiou G., Saint A., Ruff M., Rekad Z., Ciais D., van Obberghen-Schilling E. (2020). Shaping Up the Tumor Microenvironment with Cellular Fibronectin. Front. Oncol..

[264] Cox T.R., Bird D., Baker A., Barker H.E., Ho M.W., Lang G., Erler J.T. (2013). LOX-Mediated Collagen Crosslinking is Responsible for Fibrosis-Enhanced Metastasis. Cancer Res..

[265] Lampi M.C., Reinhart-King C.A. (2018). Targeting Extracellular Matrix Stiffness to Attenuate Disease: From Molecular Mechanisms to Clinical Trials. Sci. Transl. Med..

[266] Riley D.J., Kerr J.S., Berg R.A., Ianni B.D., Pietra G.G., Edelman N.H., Prockop D.J. (1982). Beta-Aminopropionitrile Prevents Bleomycin-Induced Pulmonary Fibrosis in the Hamster. Am. Rev. Respir. Dis..

[267] Yuan L., Siegel M., Choi K., Khosla C., Miller C.R., Jackson E.N., Piwnica-Worms D., Rich K.M. (2007). Transglutaminase 2 Inhibitor, KCC009, Disrupts Fibronectin Assembly in the Extracellular Matrix and Sensitizes Orthotopic Glioblastomas to Chemotherapy. Oncogene.

[268] Caron N.S., Munsie L.N., Keillor J.W., Truant R. (2012). Using FLIM-FRET to Measure Conformational Changes of Transglutaminase Type 2 in Live Cells. PLoS ONE.

[269] Kerr C., Szmacinski H., Fisher M.L., Nance B., Lakowicz J.R., Akbar A., Keillor J.W., Lok Wong T., Godoy-Ruiz R., Toth E.A. (2017). Transamidase Site-Targeted Agents Alter the Conformation of the Transglutaminase Cancer Stem Cell Survival Protein to Reduce GTP Binding Activity and Cancer Stem Cell Survival. Oncogene.

[270] Forni C., Braglia R., Mulinacci N., Urbani A., Ronci M., Gismondi A., Tabolacci C., Provenzano B., Lentini A., Beninati S. (2014). Antineoplastic Activity of Strawberry (Fragaria × Ananassa Duch.) Crude Extracts on B16-F10 Melanoma Cells. Mol. BioSyst..

[271] Kim S. (2018). New Insights into Development of Transglutaminase 2 Inhibitors as Pharmaceutical Lead Compounds. Med. Sci..

[272] Besouw M., Masereeuw R., van den Heuvel L., Levtchenko E. (2013). Cysteamine: An Old Drug with New Potential. Drug Discov. Today.

[273] Reversibly Acting Transglutaminase 2 Inhibitors: Drug Candidates for the Treatment of Fibrosis. https://zedira.com/News/Reversibly-acting-transglutaminase-2-inhibitors-drug-candidates-for-the-treatment-of-fibrosis_127.

[274] Harrison S.A., Abdelmalek M.F., Caldwell S., Shiffman M.L., Diehl A.M., Ghalib R., Lawitz E.J., Rockey D.C., Schall R.A., Jia C. (2018). Simtuzumab is Ineffective for Patients with Bridging Fibrosis rr Compensated Cirrhosis Caused by Nonalcoholic Steatohepatitis. Gastroenterology.

[275] Winer A., Adams S., Mignatti P. (2018). Matrix Metalloproteinase Inhibitors in Cancer Therapy: Turning Past Failures into Future Successes. Mol. Cancer Ther..

